# Reconstruction of *Oryza sativa indica* Genome Scale Metabolic Model and Its Responses to Varying RuBisCO Activity, Light Intensity, and Enzymatic Cost Conditions

**DOI:** 10.3389/fpls.2017.02060

**Published:** 2017-11-30

**Authors:** Ankita Chatterjee, Benazir Huma, Rahul Shaw, Sudip Kundu

**Affiliations:** Department of Biophysics, Molecular Biology and Bioinformatics, University of Calcutta, Kolkata, India

**Keywords:** *Oryza sativa indica*, metabolic model, flux balance analysis, photorespiration, enzymatic cost, light intensity

## Abstract

To combat decrease in rice productivity under different stresses, an understanding of rice metabolism is needed. Though there are different genome scale metabolic models (GSMs) of *Oryza sativa japonica*, no GSM with gene-protein-reaction association exist for *Oryza sativa indica*. Here, we report a GSM, OSI1136 of *O.s. indica*, which includes 3602 genes and 1136 metabolic reactions and transporters distributed across the cytosol, mitochondrion, peroxisome, and chloroplast compartments. Flux balance analysis of the model showed that for varying RuBisCO activity (*V*_c_/*V*_o_) (i) the activity of the chloroplastic malate valve increases to transport reducing equivalents out of the chloroplast under increased photorespiratory conditions and (ii) glyceraldehyde-3-phosphate dehydrogenase and phosphoglycerate kinase can act as source of cytosolic ATP under decreased photorespiration. Under increasing light conditions we observed metabolic flexibility, involving photorespiration, chloroplastic triose phosphate and the dicarboxylate transporters of the chloroplast and mitochondrion for redox and ATP exchanges across the intracellular compartments. Simulations under different enzymatic cost conditions revealed (i) participation of peroxisomal glutathione-ascorbate cycle in photorespiratory H_2_O_2_ metabolism (ii) different modes of the chloroplastic triose phosphate transporters and malate valve, and (iii) two possible modes of chloroplastic Glu–Gln transporter which were related with the activity of chloroplastic and cytosolic isoforms of glutamine synthetase. Altogether, our results provide new insights into plant metabolism.

## Introduction

Rice (*Oryza sativa*) is one of the major food crops cultivated worldwide and it provides nearly 20% of the caloric intake for humans. Several environmental conditions like drought, flood, nutrient availability etc. govern the rice yield. In order to understand the effect of these factors on rice production it is important to study rice cellular physiology which includes metabolism under various conditions. One of the approaches toward understanding rice metabolism is reconstruction and analysis of its genome scale metabolic model (GSM). GSMs represent a list of all reactions encoded in the genome of the target species and associated metabolites. GSMs of several plants have been reconstructed including *Arabidopsis thaliana* (Poolman et al., 2009; Mintz-Oron et al., 2012), rapeseed (Pilalis et al., 2011) and leaf, embryo and endosperm of maize (Seaver et al., 2015). The first GSM of rice (*japonica)* was reported in 2013 which was used to study rice metabolism under varying light intensity (Poolman et al., 2013). Recently, this GSM of rice was curated for analysis of chlorophyll synthesis pathway (Chatterjee and Kundu, 2015). The first metabolic/regulatory network model of rice, which represented two tissue types: germinating seeds and photorespiring leaves was reported in 2013 (Lakshmanan et al., 2013). Later it was reconstructed into a fully compartmentalized GSM of rice (iOS2164) and was used to study rice metabolism under different light treatments (Lakshmanan et al., 2015). Further details of available rice GSMs can be found in a recent review (Lakshmanan et al., 2016). Analysis of GSMs is generally performed using constraint based approaches like flux balance analysis (FBA) and flux variability analysis (FVA). FBA predicts metabolic fluxes by optimizing a given objective function (Orth et al., 2010). The commonly used objective functions are maximization of biomass (Fong et al., 2003; Schuster et al., 2008) and minimization of total flux (Poolman et al., 2009, 2013). FVA is a method which identifies alternative optimal solutions (Mahadevan and Schilling, 2003). It determines the maximum and minimum flux values for each reaction that is involved in all alternative optimal solutions.

*Indica* and *japonica* are the two-main subspecies of Asian cultivated rice and several studies have reported differences between *japonica* and *indica* at different developmental stages and conditions (Kang et al., 2006; Liu et al., 2010; He et al., 2011; Yang et al., 2014). In the past few years, advancement in bioinformatics approaches has made it possible to detect differences between *indica* and *japonica* rice at metabolomic level (Weng and Chen, 1987; Hu et al., 2014) and metabolomics is widely used to study rice diversity (Kusano et al., 2015). Although GSMs of japonica are available, there exists no GSM of *O.s. indica.* Having a metabolic model of *indica* as well, would allow rice researchers to further improve their understanding of the characteristics of these two subspecies of Asian cultivated rice at the physiological and metabolic level.

One of the strategies for increasing crop productivity is by manipulating photorespiration (Betti et al., 2016). Given the upcoming reports of the importance of photorespiration in plant protection to abiotic and biotic stress this strategy depends on the crop plant and the environmental conditions it is grown in. Studying the response of rice plants to drought stress has acquired special attention because it affects about 50% of rice productivity worldwide (Bouman et al., 2005). Amongst others, increased rates of photorespiration is one of the effects that drought has on plants (Farooq et al., 2009). Under drought, leaf stomata are closed to prevent water loss and hence CO_2_ concentration in leaves decreases. This results in inhibited photosynthesis and increased photorespiration (PR) (Cornic, 2000; Flexas et al., 2007; Bauwe et al., 2012). We performed the study of varying *V*_c_/*V*_o_ to study leaf metabolic responses for shifting from normal air (*V*_c_/*V*_o_ = 3) to stress conditions (*V*_c_/*V*_o_ < 3). Also, plant leaves are constantly exposed to different intensities of sunlight. Such variations in light intensity may cause readjustments in plant metabolism. Previous studies have simulated the dependence of photosynthetic metabolism of prokaryotes, algae and plants on light energy (Shastri and Morgan, 2005; Chang et al., 2011; Nogales et al., 2012; Poolman et al., 2013; Lakshmanan et al., 2015). These studies have shown that depending on light conditions alternative pathways may become operative to optimize photosynthetic performance. Moreover, plants are frequently exposed to rapidly changing environmental conditions which may alter the expression of genes and proteins and consequently a plant’s active metabolism. Integration of rice GSMs and transcriptomic data helps in identifying major transcriptional regulators involved in metabolic adjustments necessary for adaptation to drought (Mohanty et al., 2016). Further, using gene co-expression networks of a legume *Lotus japonicus*, it has been established that there exists a clear correlation between genes involved in photorespiration and primary nitrogen assimilation (Pérez-Delgado et al., 2016). The different enzymatic states of a plant cell can be mimicked by the method of assigning random weights to the metabolic reactions and transporters in the model (Shaw and Kundu, 2013; Cheung et al., 2015). Additionally, unlike FBA, which does not automatically identify the flux distributions through the alternative pathways within a network, random weighting makes it possible to identify different pathways that become active in individual simulations (Cheung et al., 2015).

Here, we report a GSM of *O.s. indica* (OSI1136) which represents a photosynthetic leaf tissue, with 1136 reactions, 1330 metabolites and gene protein reaction (GPR) association for 3602 unique genes. The reconstructed model was simulated and analyzed under three conditions – (i) varying flux ratio of carboxylation to oxygenation of RuBisCO (*V*_c_/*V*_o_) from 1:1 to 5:1 (ii) varying light intensity (light scan) and (iii) different enzymatic costs by assigning random weights to the reactions in the model. *V*_c_/*V*_o_ < 3 corresponds to increased rate of photorespiration which is representative of drought stress in plants (Wingler et al., 1999; Cornic, 2000; Flexas et al., 2007; Bauwe et al., 2012), *V*_c_/*V*_o_ = 3 represents photorespiration under normal air conditions (Jordan and Ogren, 1984) and *V*_c_/*V*_o_ > 3 represents increased activity of RuBisCO carboxylase and decreased photorespiration (Jordan and Ogren, 1984). Besides validating some of the results with previously reported experimental evidences and model simulation studies, we have provided some new insights into plant cellular metabolism under the above mentioned conditions. The study shows that the glyceraldehyde-3-phosphate dehydrogenase (GAPDH) and phosphoglycerate kinase (PGK) mediated reactions act as source of cytosolic ATP needed by plants under decreased photorespiration and their activities are interlinked with the rate of mitochondrial ATP generated. Further, the activity of the chloroplastic malate valve was found to increase suggesting its possible role in transporting reducing equivalents out of the chloroplast under increased PR conditions which is likely during drought stress. Next, we found that for different ranges of light intensity, the flux values of the chloroplast triose phosphate transporters (TPTs), mitochondrial ATP transporter, dicarboxylate transporters of the chloroplast and the mitochondrion increased, decreased or showed complete inactivity to meet cellular ATP and NAD(P)H demands in plants. Given any particular light range, the transporters that are active or their fluxes differed from other ranges. Variations in the participation of the glutathione-ascorbate cycle; chloroplastic TPTs and the malate valve were observed under different enzymatic costs. We further observed that, the chloroplastic Glu–Gln transporter could operate reversibly to either export or import Glu. Consequently, it was related with the activity of chloroplastic and cytosolic isoforms of glutamine synthetase (GS), i.e., GS2 and GS1, respectively. Altogether, the reconstructed model of *O.s. indica* with given GPR can serve to understand rice metabolism and it’s interlink with other cellular processes.

## Materials and Methods

### Metabolic Reconstruction

The GSM of *O.s. indica* was reconstructed starting with its protein sequences available in the NCBI protein database. The protein sequences were subjected to PRIAM (Claudel-Renard et al., 2003). PRIAM allows automated enzyme detection by mapping the query sequence against PRIAM profiles. It has been used on several bacterial species (Fleischmann et al., 1995; Blattner, 1997; Shigenobu et al., 2000) and also, on oilseed species *Ricinus communis* (Chan, 2010). EC numbers with hits that showed *E* value < 10^-10^ were considered (Chan, 2010) and were mapped against METACYC database (Caspi et al., 2007) to obtain the corresponding reactions. This gave us the first draft model with 1101 reactions. Next, the draft model so formed consisted of several generic reactions that involved substrates like acceptors, peptides, ssRNAs, mRNAs and tRNAs. So, based on literature search and database like BRENDA (Schomburg et al., 2004) the draft model was manually curated to check for the generic reactions followed by removal of several non-plant reactions. 206 such reactions were removed from the draft model (Supplementary Table S1).

### Gap Filling and Compartmentalization

Removal of non-plant reactions was followed by addition of some reactions. Many reactions that are known to be involved in the synthesis of biomass components in plants were found to be missing from the model. Therefore, we incorporated the missing reactions in the draft model based on literature study, databases like Metacyc and Brenda and other plant models (Poolman et al., 2009; Chatterjee and Kundu, 2015). For example, the reactions involved in the last step of lysine synthesis (diaminopimelate decarboxylase, 4.1.1.20) and methionine synthesis (methionine synthase, 2.1.1.14) were absent in the draft model, hence these reactions were included. Additionally, we also added the reactions needed for flavin adenine dinucleotide (FAD) and flavin mononucleotide (FMN) synthesis. In all, 125 reactions were added to the model (Supplementary Table S2). Next, we aimed at localization of the individual reactions in the draft model to appropriate subcellular compartments based on literature evidences and available plant models (Poolman et al., 2009; Chatterjee and Kundu, 2015). Here, we considered four compartments- cytosol, mitochondrion (‘mit_’), chloroplast (‘chl_’) and peroxisome (‘per_’). Reactions associated with TCA cycle and ETC were put into mitochondrion module. Additionally, we added one transporter for proton and one for ATP (Nelson et al., 2008). Reactions for Calvin cycle, glycolysis, starch metabolism, cyclic and non-cyclic photophosphorylation, nitrogen assimilation and chlorophyll synthesis were put in the chloroplast module. For the peroxisome module, we considered reactions involved with glutathione-ascorbate cycle (Jimenez et al., 1997). Reactions associated with photorespiration were distributed in the chloroplast, mitochondrion and peroxisome (Bauwe et al., 2010). Further, transport reactions were also defined and were represented using the subscript ‘_tx’. Transport reactions were added to produce essential biomass precursors from inorganic nutrients consisting of water, CO_2_, oxygen, nitrogen as NH_3_ and NO_3_, phosphate, sulfate, and photons. At this stage, we obtained a model which consisted of 1136 reactions and 1330 metabolites.

### Biomass Composition

All the biomass components except chlorophyll and nucleic acids were considered in a proportion as provided by Juliano (1986). The chlorophyll and the nucleic acids contents were estimated from Kwon and Soh (1985); Maruyama et al. (1990), respectively. We assigned individual transporters for each biomass components and the flux through each of them was set independently in experimentally fixed proportions (Supplementary Data Sheet S3). Please see Supplementary Data Sheet S1 for detail description of Supplementary Information.

### Model Analysis

#### Flux Balance Analysis

The model was checked for mass, energy and redox conservations and does not have any stoichiometric inconsistencies (Poolman et al., 2006, 2013; Gevorgyan et al., 2008). The model was analyzed using FBA (Lee et al., 2006). FBA considers a system to be at steady state in which the rate of production of each internal metabolite in the network is equal to its rate of consumption. This state can be represented mathematically as:

S.v=0

where *v* is a vector of fluxes through the metabolic network and *S* is the stoichiometry matrix (Lee et al., 2006). *S* is an m × n matrix where *m* represents the rows and the corresponding metabolites and *n* represents the columns and the corresponding reactions. We used Gnu Linear Programming Kit^1^ and linear programming (LP) based ScrumPy metabolic modeling package (Poolman, 2006). The LP was defined as:

minimize⁢ Z=c.v

subject⁢ to⁢ S.v=0

LB≤v≤UB

Here, *Z* is the objective function, *v* is the flux vector, *c* is the transpose of a vector of objective coefficients, LB and UB are the vectors of fluxes lower bounds and upper bounds, respectively. The objective function used in the analysis was minimization of the total cellular flux (sum of absolute flux values carried by all the reactions). The biomass transporters were set to carry fixed flux (Supplementary Data Sheet S3) such that the biomass components can be produced in experimentally defined proportions. Light energy, water, CO_2_ as the sole carbon source and other inorganic nutrients like nitrogen as NH_3_ and NO_3_, sulfate, and phosphate were supplied as substrates to enable biomass synthesis. The flux of the nutrients and the photon flux (*v_v_*) were left unconstrained, unless specified. A cell maintenance cost was assumed to 0.1 flux unit through a generic ATPase reaction (Poolman et al., 2013).

#### Flux Variability Analysis

To find the allowable range of flux of each of the reactions under drought like and normal air conditions (*V*_c_/*V*_o_ = 1 and *V*_c_/*V*_o_ = 3 respectively), FVA was performed. FVA calculates the maximum and minimum allowable flux values of each of the reactions of the solution space, while satisfying the optimality of a given objective function. The optimal objective value of the primary objective function (here, the sum of flux values) was computed for synthesis of all the biomass components in experimentally defined proportions. For each reaction in the solution space, the reaction flux was maximized and minimized to get the allowable range of flux values.

### Light Scan

In addition to the above-mentioned constraints, the photon flux (*v_v_ = v*) was varied, starting at the point at which solution was obtained till changes in reaction-fluxes remained linear. Thus, the photon flux was varied from 0.33 to 10.00 flux unit. The lower limit indicated the photon flux below which there existed no feasible solution for biomass synthesis under defined constraints. The upper limit indicated the photon flux beyond which the flux plot of the reactions remained uniform. An additional constraint was that the flux of cyclic photophosphorylation could not exceed that of non-cyclic.

### Photorespiratory Metabolism

We simulated the photorespiratory metabolism by setting the carboxylation to oxygenation flux ratio (*V*_c_/*V*_o_) of RuBisCO from 1:1 to 5:1. The RuBisCO catalyzed carboxylation and oxygenation reactions were combined into a single reaction according to the *V*_c_/*V*_o_ investigated (Supplementary Data Sheet S3). *V*_c_/*V*_o_ = 3 represents photorespiration under normal air conditions (Jordan and Ogren, 1984), *V*_c_/*V*_o_ < 3 corresponds to increased photorespiration under drought conditions (Wingler et al., 1999) and *V*_c_/*V*_o_ > 3 represents increased activity of RuBisCO carboxylase and decreased photorespiration (Jordan and Ogren, 1984).

### Implementing the Enzymatic Cost through Random Weighting

The method of assigning random weights to the reactions (Shaw and Kundu, 2013) has been used in model analysis (Cheung et al., 2015) and was implemented in this study. We set the objective function as minimization of the total cellular flux. Under varying conditions, cells adapt to different metabolic states because of the changes in the gene expression of enzymes. Therefore, in order to mimic gene expression of enzymes, random weights (from 0 to 1000) were assigned to all the reactions except the transporters and light cyclic and non-cyclic photophosphorylation. Further, nutrients uptake and photon flux were free to use any value (≥0). We simulated the metabolic responses for 10000 iterations. Each of the iteration generated a possible solution for biomass synthesis under different enzymatic cost conditions.

## Results and Discussion

### Model Properties

We reconstructed the GSM of *O.s. indica* with gene protein reaction association based on its protein sequences. The final GSM, OSI1136 consists of 1136 reactions and 1330 metabolites localized across the cytosol, chloroplast, mitochondrion and peroxisome compartments. We mapped the protein sequences against Uniprot (UniProt Consortium, 2015) and obtained a list of 3602 genes and associated reactions. Supplementary Data Sheet S2 provides the GPR association for the reactions present in the model. Details of the model can be found in Supplementary Data Sheet S3. There are 943 metabolic reactions in the cytosol, 17 in mitochondrion, 70 in chloroplast, and 14 in peroxisome. The model also includes 48 intracellular transporters and 44 biomass transporters. The model is capable of producing biomass precursors (amino acids, starch, nucleic acids, lipids, glucose, sucrose, cellulose, lignin, and chlorophyll a) in experimentally fixed proportion using light energy, CO_2_ as the sole carbon source and other inorganic nutrients like nitrogen, sulfate, and phosphate.

### Comparison with Other GSM Models of Rice

**FIGURE 1:**
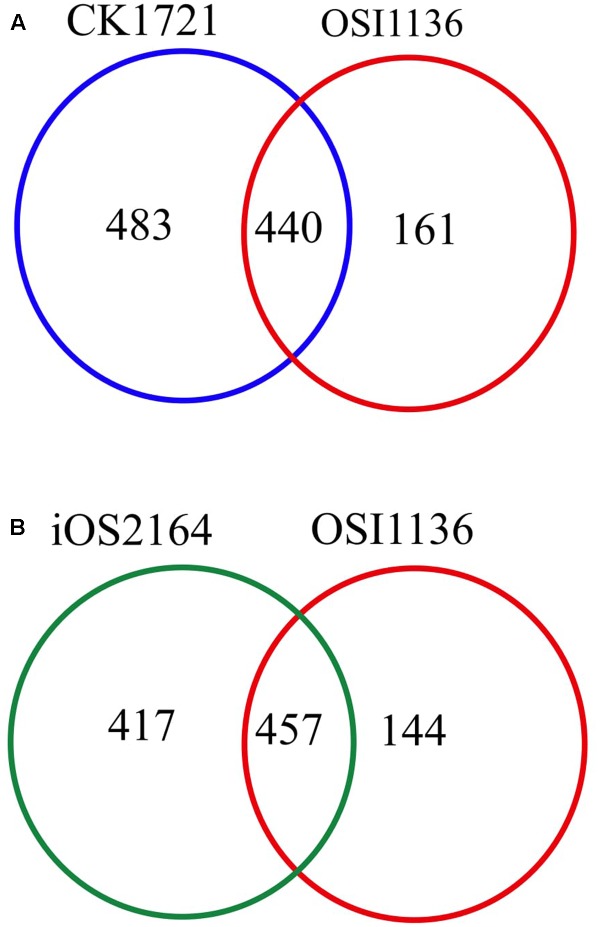
Venn diagram showing comparison of the EC numbers corresponding to the respective reactions present in genome scale metabolic models (GSM) of *Oryza sativa japonica* and *Oryza sativa indica*. **(A)** Comparison of EC numbers associated with CK1721 (Chatterjee and Kundu, 2015) and OSI1136. **(B)** Comparison of EC numbers associated with iOS2164 (Lakshmanan et al., 2015) and OSI1136.

The first GSM of rice leaf (*O.s. japonica*) was reported in 2013 (Poolman et al., 2013). Although the model was able to predict some of the physiological behavior of plants, it lacked some important aspects related to plant metabolism, such as, the chlorophyll synthesis pathway and the peroxisome compartment. Recently, the model was curated and updated by adding the chloroplast specific reactions for chlorophyll synthesis, the peroxisome compartment, and by removing some non-plant reactions (Chatterjee and Kundu, 2015; here we refer to this model as CK1721). To reconstruct the model for *indica* we started with its protein and we used the updated rice *japonica* model (CK1721) as a reference for gap filling. Recently, a rice *japonica* model (iOS2164) was reported (Lakshmanan et al., 2015) which consists of additional compartments namely, endoplasmic reticulum (ER), vacuole, and thylakoid. We compared the EC numbers corresponding to the respective reactions present in OSI1136 with CK1721 and iOS2164. We did not compare the reactions by their names in order to avoid any discrepancy in case of same reactions represented by different names. There are 923 and 601 EC numbers associated with CK1721 and OSI1136 respectively (excluding transport reactions). 440 EC numbers and the corresponding reactions (701 reactions) were common in CK1721 and OSI1136, while 161 EC numbers and the corresponding reactions (343 reactions) were unique in OSI1136 (**Figure 1A** and **Table 1**). These unique reactions are mainly involved in secondary metabolism such as jasmonic acid synthesis pathway (for example, ALLENE-OXIDE-SYNTHASE-RXN, ALLENE-OXIDE-CYCLASE-RXN, 12-OXOPHYTODIENOATE-REDUCTASE-RXN, RXN-1501), phytoalexin synthesis pathway (for example, RXN-4882, RXN-4883, RXN-8528, RXN-4881) fatty acid beta oxidation (for example, 5.1.2.3-RXN, 2.6.1.80-RXN, RXN-6383) etc. (Supplementary Data Sheet S4). Jasmonic acid synthesis is known to play role in plant development and responses to environmental stresses, such as mechanical wounding or pathogen attack (León and Sánchez-Serrano, 1999). Phytoalexins possess antimicrobial activity toward phytopathogens (Cho and Lee, 2015) and have been seen to accumulate in plants in response to microbial infection or abiotic stress (Kuc, 1995). Having these reactions included in the model would enable future *in silico* investigation of rice secondary metabolism. In comparison with iOS2164, 144 EC numbers were unique in OSI1136 (**Figure 1B**). Compared to iOS2164 the *indica* model has lesser number of reactions firstly, because the former has considered reactions associated with the thylakoid, vacuole and ER compartments. Secondly, instead of including all the steps of cyclic and non-cyclic photophosphorylation (as considered in iOS2164), in OSI1136, we represented them as single reactions which were obtained from elementary modes analysis (Poolman et al., 2013).

**Table 1:** Comparison of number of reactions present in GSM of *Oryza sativa japonica* and *Oryza sativa indica.*

	GSM of *O.s. indica* (OSI1136)	GSM of *O.s. japonica* (CK1721)	GSM of *O.s. japonica* (iOS2164)
Cytosol	943	1576	893
Mitochondria	17	19	236
Chloroplast	70	72	556
Peroxisome	14	5	84
No. of biomass transporters	38	1	45
No. of intracellular transporters	46	40	309
No. of extracellular transporters	8	8	15
Others	–	–	160
Total No. Of Reactions	1136	1721	2283
Total No. Of metabolites	1330	1544	1999


### Metabolic Responses to Varying *V*_c_/*V*_o_

Photorespiration is induced by drought stress primarily because of the stomatal closure and restricted diffusion of CO_2_ into the leaf (Quick et al., 1992). Labeling studies had showed that scarcity of water accelerates the photorespiratory pathway (Lawlor, 1976; Lawlor and Pearlman, 1981). A study also reported that plants showed increased photorespiration under drought stress in order to adapt to excess light energy absorbed (Smirnoff, 1993). Although drought stress adaptation is related to complex regulatory and signaling effects (Harb et al., 2010; Basu et al., 2016; Deb et al., 2016), our efforts were confined to photorespiratory and related metabolism under drought like stress conditions. We simulated the reconstructed OSI1136 from *V*_c_/*V*_o_ = 1 to *V*_c_/*V*_o_ = 5 and report its metabolic responses under increased PR, PR in normal air conditions and decreased PR.

### Variation in ATP/NAPDH Ratio and Quantum Demand

**FIGURE 2:**
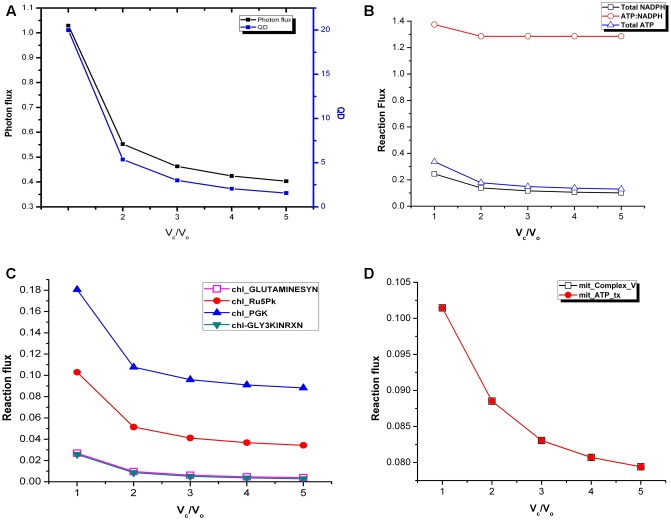
Variations in photon flux, quantum demand and ATP:NADPH ratio under varying *V*_c_/*V*_o_. **(A)** The photon flux and the QD were maximum at *V*_c_/*V*_o_ = 1. **(B)** The total ATP and NADPH generated were maximum and minimum at *V*_c_/*V*_o_ = 1 and *V*_c_/*V*_o_ = 5, respectively. ATP:NADPH ratio was found to be same from *V*_c_/*V*_o_ = 2 to *V*_c_/*V*_o_ = 5. **(C)** GS2, chl_Ru5Pk, chl_PGK, and GLYK carried maximum flux at *V*_c_/*V*_o_ = 1 and decreased thereafter. **(D)** The ATP generation through mitochondrial Complex *V* and its release by the ATP transporter was maximum at *V*_c_/*V*_o_ = 1. The reactions present in the chloroplast and mitochondrion are represented by chl_ and mit_ respectively. _tx represents transport reactions.

We calculated the photon flux needed to produce ATP and NADPH which ultimately are required for biomass synthesis in fixed proportion and cellular maintenance. There was a decrease in photon flux from *V*_c_/*V*_o_ = 1 to *V*_c_/*V*_o_ = 5 (**Figure 2A**). At the same time fluxes of cyclic and non-cyclic photophosphorylation showed that the total ATP and NADPH generated was maximum at *V*_c_/*V*_o_ = 1 and minimum at *V*_c_/*V*_o_ = 5 (**Figure 2B**). Despite the changes in total ATP and NADPH, the ATP:NADPH ratio was found to be same from *V*_c_/*V*_o_ = 2 to *V*_c_/*V*_o_ = 5 (**Figure 2B**). While the non-cyclic photophosphorylation was always active the cyclic photophosphorylation carried flux only at *V*_c_/*V*_o_ = 1. The higher amount of ATP generated in the chloroplast at *V*_c_/*V*_o_ = 1 was found to be partly used in photorespiratory ammonia fixation in the chloroplast (by chl_GLUTAMINESYN, EC 6.3.1.2) and the rest of it got utilized in the recycling of glycolate (via glycerate kinase reaction, chl_GLY3KINRXN) which is formed from phosphoglycolate (Pgly) produced in the RuBisCO oxygenase catalyzed reaction. Accordingly, the flux of these reactions was maximum at *V*_c_/*V*_o_ = 1 (**Figure 2C**). Most of the other ATP consuming reactions in the chloroplast did not show variation in the flux except chl_Ru5Pk (EC 2.7.1.19) and chl_PGK (EC 2.7.2.3). The former is the source of ribulose bisphosphate (RuBP) in the chloroplast which participates in RuBisCO carboxylase/oxygenase reaction to generate 3-phosphoglycerate (3-PGA or PGA). PGA in turn gets phosphorylated to 1,3-bisphosphogycerate (BPGA) by phosphoglycerate kinase (PGK). Both RuBP generation (by chl_Ru5Pk) and phosphorylation of PGA to BPGA (by chl_PGK) were found to be maximum to sustain the C3 cycle and to synthesize the same amount of biomass under limited carbon conditions at *V*_c_/*V*_o_ = 1 (**Figure 2C**). The other major source of ATP (besides cyclic and non-cyclic photophosphorylation) is the mitochondrial ATP synthase (Complex_V) which also carried maximum flux at *V*_c_/*V*_o_ = 1 (**Figure 2D**). Thus, the ATP generated in the mitochondrion by Complex V and ATP released by the mitochondrial ATP transporter to cytosol was highest at *V*_c_/*V*_o_ = 1. This was because, while chloroplastic ATP met increased PR demands, ATP demand for rest of cellular metabolism was met by mitochondrial ATP. In addition, we also calculated the quantum demand (photon needed per CO_2_ fixed; QD) from *V*_c_/*V*_o_ = 1 to *V*_c_/*V*_o_ = 5. As expected, the fall in the QD from *V*_c_/*V*_o_ = 1 to *V*_c_/*V*_o_ = 5 was similar to that observed for photon flux (**Figure 2A**). The higher photon flux ultimately reflects the requirement to synthesize the same amount of biomass under limited carbon conditions where the efficiency of photosynthetic triose phosphate production reduces and where carbon recycling by photorespiration becomes primary.

### Enhanced Activity of the Malate Valve at *V*_c_/*V*_o_ < 3

**FIGURE 3:**
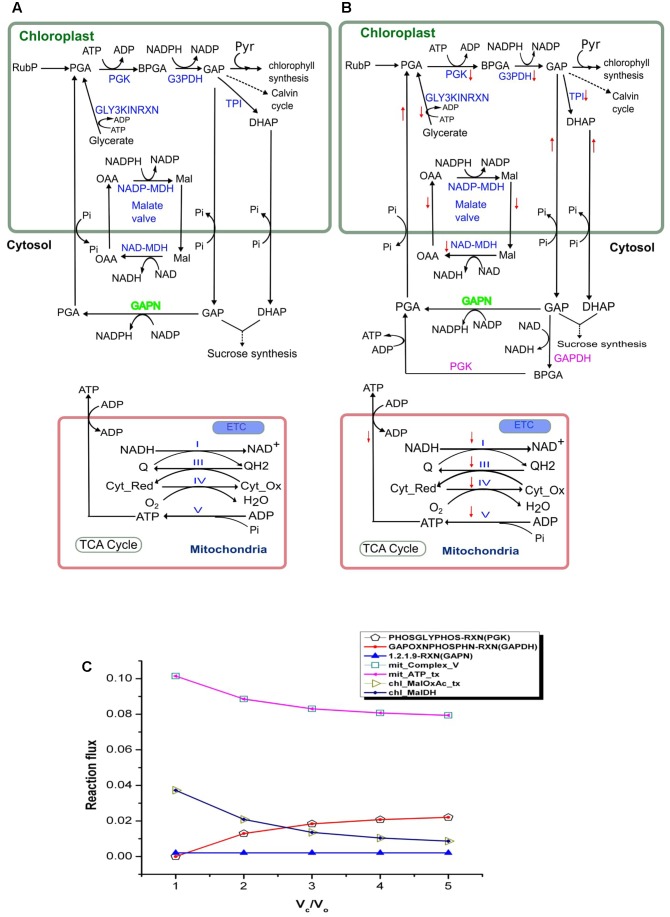
Activity of the chloroplastic malate valve, cytosolic GAPDH and PGK and mitochondrial electron transport chain (ETC) at *V*_c_/*V*_o_ = 1 and *V*_c_/*V*_o_ = 3. Activity of the chloroplastic malate valve, cytosolic GAPDH, PGK and mitochondrial ETC at **(A)** *V*_c_/*V*_o_ = 1 and **(B)** normal air condition, *V*_c_/*V*_o_ = 3. Reactions names in blue indicate the reactions which showed variation in flux; reaction names in green indicate reactions which showed no variation in flux; reaction names in pink are the ones which were not active at *V*_c_/*V*_o_ = 1. Down arrow in red represents decrease in flux of the respective reaction. The flux through the chloroplastic triose phosphate transporters (TPT; ‘chl_PGA_tx’, ‘chl_GAP_tx’, and ‘chl_DHAP_tx’) increased at *V*_c_/*V*_o_ = 3. **(C)** Flux plot for reactions catalyzed by PGK (PHOSGLYPHOS -RXN), GAPDH (GAPOXNPHOSPHN -RXN), GAPN (1.2.1.9-RXN), chloroplastic malate dehydrogenase (chl_MalDH), chloroplastic malate-oxaloacetate shuttle (chl_MalOxAc_tx), mitochondrial complex V (mit_Complex_V), and mitochondrial ATP transporter (mit_ATP_tx). The mit_Complex_V, mit_ATP_tx, PHOSGLYPHOS –RXN, GAPOXNPHOSPHN-RXN, chl_MalDH, and chl_MalOxAc_tx carried varying flux while GAPN carried the same flux. PGA, 3-phosphoglycerate; BPGA, 1,3-bisphosphoglycerate; Mal, malate; OAA, oxaloacetic acid; Pyr, pyruvate; GAP, glyceraldehyde-3-phosphate; Cyt_red, reduced cytochrome; Cyt_Ox, oxidized cytochrome. PGK, phosphoglycerate kinase (EC 2.7.2.3); G3PDH, glyceraldehyde-3-phosphate dehydrogenase (EC 1.2.1.13); GAPN, glyceraldehyde-3-phosphate dehydrogenase (NADP+) (EC 1.2.1.9); GAPDH, glyceraldehyde-3-phosphate dehydrogenase (EC 1.2.1.12); TPI, triose-phosphate isomerase (EC 5.3.1.1); NADP-MDH, NADP-dependent malate dehydrogenase (EC 1.1.1.82); NAD-MDH, NAD-dependent malate dehydrogenase (EC 1.1.1.37).

Plant membranes are broadly impermeable to NAD(P) and NAD(P)H, so they possess specific transporters for the exchange of Mal and OAA which enable the indirect transport of reducing equivalents between different cellular compartments. NADPH dependent malate dehydrogenase (NADP–MDH) in the chloroplast converts OAA to Mal, facilitating the regeneration of the electron acceptor NADP in the chloroplasts. The enzyme (MDH) is known to play an important role in maintaining redox balance in plants under changing environmental conditions (Scheibe et al., 2005). The ‘malate valve’ (combined activity of Mal–OAA transporter and NADP–MDH) acts as a powerful system for balancing the ATP/NAD(P)H ratio in plants (Scheibe, 2004). We observed that under drought stress (*V*_c_/*V*_o_ < 3) when the chloroplastic NADPH generation is high (as described in the previous section) the activities of Mal-OAA transporter and NADP–MDH were also high; and with decrease in photorespiration (i.e., increased CO_2_ assimilation) the flux through the Mal–OAA and NADP–MDH decreased (**Figure 3**). The observation suggested that the malate valve is activated to transport excess reducing equivalents out of chloroplasts under drought stress and at higher rates of photorespiration. This observation was supported by a previous experiment on tobacco plants which reported reduced expression of NADP–MDH at decreased rate of photorespiration (Backhausen and Scheibe, 1999).

### GAPDH and PGK Can Act as a Source of Cytosolic ATP under Decreased Photorespiration

The glycolytic pathway in plant’s cytosol consists of alternative ways to metabolize GAP to PGA. One way occurs via the GAPDH (EC 1.2.1.12) and phosphoglycerate kinase (PGK, EC 2.7.2.3) generating NADH and ATP; the second is a single step catalyzed by non-phosphorylating glyceraldehyde-3-phosphate dehydrogenase (EC 1.2.1.9; GAPN; or NP-GAPDH) and generating NADPH (Rius et al., 2006). The study of NP-GAPDH mutant had revealed a significant role of this enzyme in plant growth and development (Rius et al., 2006). However, a more precise role of both the pathways in metabolism under stress conditions like drought has not been explored. Here, we specifically studied the changes in the activity of both the pathways under varying *V*_c_/*V*_o_.

The analysis of the model simulation showed that GAPN was operative and carried same flux throughout varying *V*_c_/*V*_o_ (**Figure 3**). However, GAPDH and PGK were inactive at *V*_c_/*V*_o_ = 1 (**Figure 3A**), active at *V*_c_/*V*_o_ = 3 (**Figure 3B**) and the flux through these reactions increased from *V*_c_/*V*_o_ = 2 to *V*_c_/*V*_o_ = 5 (**Figure 3C**). The export of GAP from the chloroplast increased at *V*_c_/*V*_o_ = 3 which was used in GAPN and GAPDH. At the same time the flux through the mitochondrial Complex V decreased. Consequently, the ATP generation in the mitochondrion and its release decreased (**Figure 3B**). Under this condition the need of ATP and NADH in the cytosol were fulfilled by the combined activity of GAPDH and PGK. On the other hand, the ATP generated in the mitochondrion by Complex V was maximum at *V*_c_/*V*_o_ = 1 (**Figure 3C**). Thus, the mitochondrion serves to play an important role in supplying the cytosolic ATP under drought stress as discussed earlier. Cytosolic GAPDH and malate valve are important for maintaining cellular redox homeostasis in plants (Hebbelmann et al., 2011). Maintenance of both redox and ATP balance is an important factor in driving plant metabolism under different conditions as such we observed that under photorespiratory conditions malate valve while under non-photorespiratory conditions GAPDH operated to supply the cytosol with chloroplastic redox.

Flux variability analysis was performed to examine the flux capacity for each reaction under the simulated conditions (Supplementary Data Sheet S5). In our analysis, the minimization of the total flux values of the reactions was set as the primary objective and the biomass transporters carried fixed flux. The metabolism was simulated under drought stress (*V*_c_/*V*_o_ = 1) and normal air condition (*V*_c_/*V*_o_ = 3). The minimum and maximum flux obtained from FVA (FVA_min_ and FVA_max_, respectively) for chloroplastic Mal-OAA shuttle and malate dehydrogenase showed decrease in their activities at *V*_c_/*V*_o_ = 3 compared to *V*_c_/*V*_o_ = 1 which supported our results of FBA (**Figure 3**). Also, the flux range for GAPDH and PGK catalyzed reactions showed that at *V*_c_/*V*_o_ = 1 both the reactions carried zero flux and were operative at *V*_c_/*V*_o_ = 3. This also supported our FBA predictions as described in **Figure 3**. While some reactions like chl_61118RXN, chl_DXSRXN, HISTALDEHYD-RXN showed FBA_val_ = FVA_min_ = FVA_max_ (FBA_val_ refers to the flux value obtained from FBA), other reactions like mit_AconDHatase, mit_AconHydr etc. always showed FBA_val_ = FVA_min_ < FVA_max_ (Supplementary Data Sheet S5). Both of these results indicated that these reactions were essentially required for biomass synthesis. For rest of the reactions it was observed that FVA_min_ < FBA_val_ < FVA_max_. The reactions with negative and positive values of FVA_min_ and FVA_max_, respectively indicated that such reversible reactions could operate in both directions. Further, FVA_min_ = 0 and FVA_max_ ≥ 0 for some reactions suggested presence of alternate metabolic routes.

### Effect of Varying *V*_c_/*V*_o_ on the Calvin Cycle and the TCA Cycle

**FIGURE 4:**
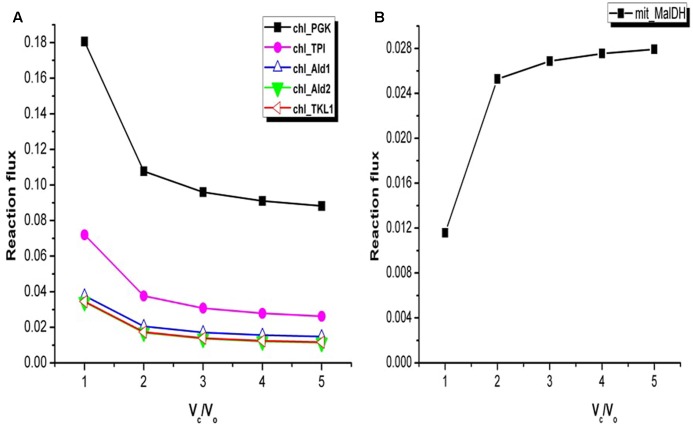
Variations in the flux of some of the reactions associated with Calvin cycle and mitochondrial malate dehydrogenase. **(A)** At *V*_c_/*V*_o_ = 1 fluxes of reactions associated with Calvin cycle were maximum. Change in fluxes of some of the Calvin cycle reactions are shown here. chl_ represents the chloroplastic reactions. **(B)** Flux of mitochondrial malate dehydrogenase (mit_MalDH) decreased from *V*_c_/*V*_o_ = 5 to *V*_c_/*V*_o_ = 1. PGK, phosphoglycerate kinase; TPI, triosephosphate isomerase; Ald1, aldolase 1; Ald2, aldolase 2; TKL1, transketolase 1.

The TCA cycle and the Calvin cycle form a part of the central metabolic pathways in plants. The flux analysis showed increase in the flux through the Calvin cycle under stress condition. For example, variations in the fluxes through some of the reactions associated with Calvin cycle are shown in **Figure 4A**. This was expected as it is required in order to support biomass synthesis in fixed proportion (Yuan et al., 2016). Only a part of the TCA cycle was found to be operative from *V*_c_/*V*_o_ = 1 to *V*_c_/*V*_o_ = 5 (the reactions catalyzed by alpha-ketoglutarate dehydrogenase, succinate thiokinase, and fumarase were always inactive). Non-cyclic modes of TCA cycle can be operative if the demand for ATP is low or if alternative sources of ATP are available (Sweetlove et al., 2010; Araujo et al., 2012). Flux analysis of *Arabidopsis* metabolism demonstrated that a cyclic TCA cycle flux is only established when the demand for ATP increases (Poolman et al., 2009). Here, the activity of the mitochondrial malate dehydrogenase (MalDH) decreased with increase in photorespiration (**Figure 4B**). Glycine oxidation and MalDH are sources of mitochondrion NADH. The flux through part of TCA cycle which operated to support biomass synthesis remained constant. As such any fluctuation in mitochondrion ATP production was associated with changes in MalDH and GDC activity. Although MalDH activity was minimum at *V*_c_/*V*_o_ = 1, the flux of mitochondrion complex V was maximum (**Figure 2D**). Thus, under such conditions photorespiratory glycine oxidation was important for ATP supply.

### Interlink in the Activities of the Chloroplastic Transporters

**FIGURE 5:**
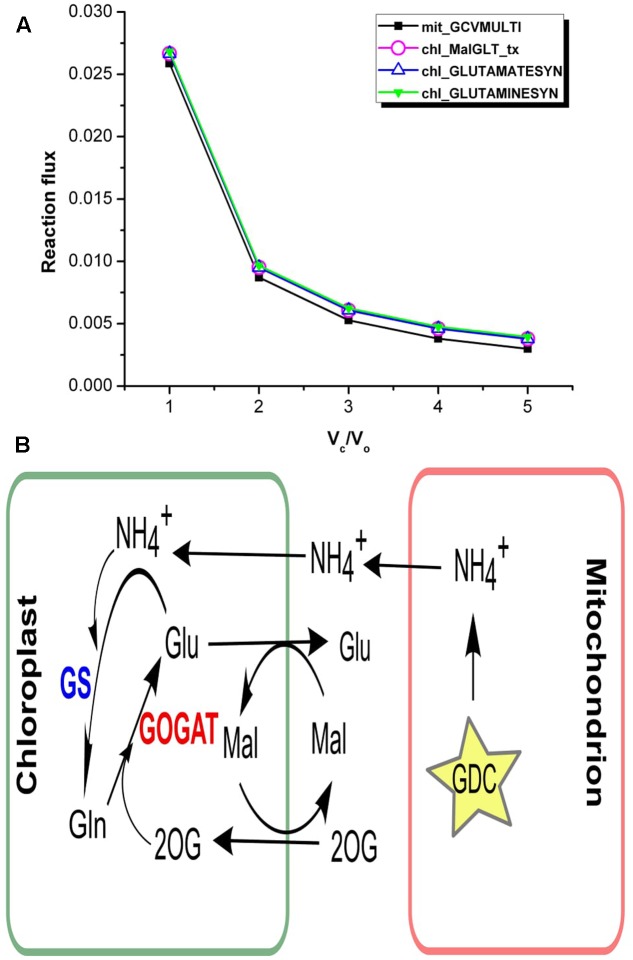
Interlink in the activities of chloroplastic transporters. **(A)** Flux plots for mitochondrial GDC catalyzed reaction (mit_GCVMULTI), GS-GOGAT cycle (chl_GLUTAMINESYN and chl_GLUTAMATESYN, respectively) and chl_MalGLT_tx. **(B)** The GS/GOGAT pathway and associated Mal-Glu and Mal-2OG transporters in plants. Photorespiratory ammonia is completely utilized in GS catalyzed reaction in the chloroplast. GDC, glycine decarboxylase; GS, glutamine synthetase; GOGAT, glutamate synthase; Mal, malate; 2OG, 2ketoglutarate; Glu, glutamate; Gln, glutamine.

*V*_c_/*V*_o_ = 1 corresponds to increased photorespiration, accordingly our results showed increase in the ammonia generated in the mitochondrion via glycine decarboxylase (GDC) (**Figure 5A**). The analysis of flux values through ammonia transporters showed that an equivalent amount of mitochondrial ammonia released into the cytosol got imported into the chloroplast where it was completely utilized in the GS/GOGAT pathway (**Figure 5B**). It is well established that the photorespiratory ammonia generated in the mitochondrion is transported to plastids, which is then re-assimilated into glutamate by GS and GOGAT (Miflin and Lea, 1980; Atkin and Macherel, 2009; Lakshmanan et al., 2013; Chatterjee and Kundu, 2015). Accordingly, the malate-2ketoglutarate (Mal-2OG) and malate-glutamate (Mal-Glu) transporters of the chloroplast were also involved (**Figure 5B**).

### Dynamic Interplay between Compartments and Reaction Interchange for Varying *V*_c_/*V*_o_

The chloroplast is the site of cyclic and non-cyclic photophosphorylation for ATP and NADPH generation. However, depending on environmental conditions metabolic adjustments particularly in mitochondrial metabolism becomes important in maintaining cellular ATP and redox homeostasis (Raghavendra and Padmasree, 2003). Although at *V*_c_/*V*_o_ = 1 both chloroplastic ATP and NADPH were maximum (**Figure 2B**), the cellular ATP demand was fulfilled mainly through mitochondrial glycine oxidation. Concomitantly, ammonia generated by glycine oxidation was refixed by GS2. Refixation of ammonia, the glycerate kinase reaction (photorespiratory metabolism) and necessary recycling of RuBP utilized the excess chloroplastic ATP (**Figures 2C**, **4D**). The excess reductant was transported out to the cytosol by the malate valve; a part of this excess reductant was used in HPR1 to sustain PR and the rest was utilized to support other cellular processes. Thus, at *V*_c_/*V*_o_ = 1 we observed coordinated metabolism wherein PR served as a sink for excess chloroplastic ATP and redox. At *V*_c_/*V*_o_ = 3, ATP generation in the mitochondrion and its release to the cytosol decreased (**Figure 3B**). Simultaneously, the export of GAP from the chloroplast increased which was used in GAPN and GAPDH. Under this condition the need of ATP and NADH in the cytosol were fulfilled by the combined activity of GAPDH and PGK. Such plasticity in plant metabolic network across different cell organelles is useful in maintaining cellular ATP and NAD(P)H homeostasis, further discussed below.

### Metabolic Responses to Varying Light Intensity

Photosynthetic metabolism is light dependent. Studies have reported that varying light conditions affect gene expression and therefore enzyme activity (Bohne and Linden, 2002). In-silico studies have successfully predicted light-driven effects on metabolism in algae (Chang et al., 2011) and cyanobacteria (Nogales et al., 2012). In this study, the RuBisCo Carboxylase and Oxygenase reactions were represented separately in the model. It was observed that with increasing light, photorespiratory metabolism became active beyond the light range 0.33–0.73.

### Dynamic Interplay of Four Compartments to Maintain Redox and ATP Balance with Increasing Photorespiration

**FIGURE 6:**
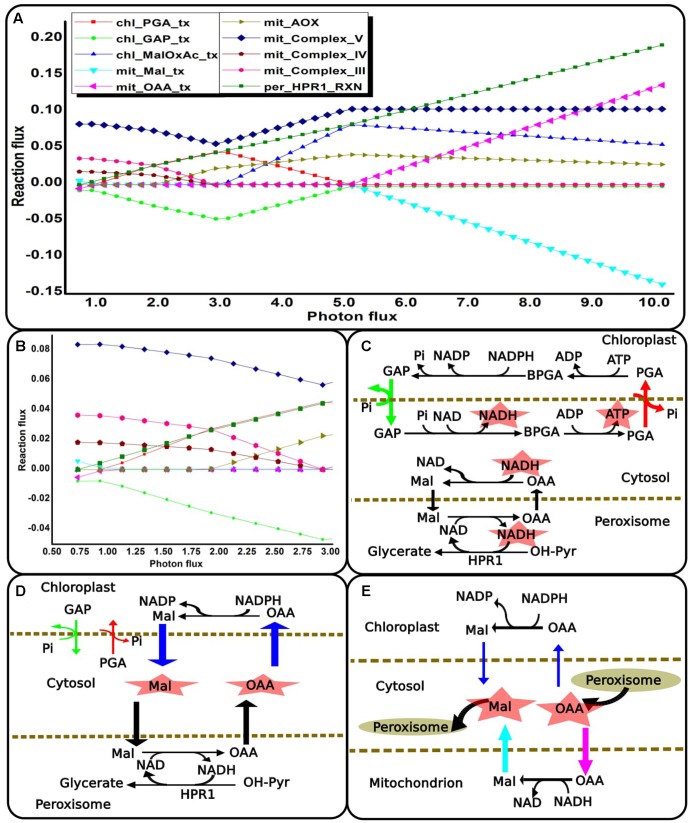
Metabolic readjustments to maintain redox and ATP balance with increasing photorespiration. **(A)** Flux-plot for reactions and transporters that showed co-ordinated changes that occurred across the light range examined. A negative flux-value of intracellular transporter implies that the metabolite is exported to the cytosol compartment. **(B)** Enlarged flux-plot for low light intensity (0.73–3.0). **(C–E)** Metabolic networks corresponding to the flux-plot shown in **(A).** These show different ways in which the NADH demand of HPR1 can be fulfilled while maintaining cellular ATP balance. The reactions shown in color correspond to their flux-plot. The differences in arrow-width represent changes in respective reaction-fluxes and transporter-fluxes. ‘chl_’ and ‘mit_’ represent reactions in the chloroplast and the mitochondrion. ‘_tx’ represents transporters. HPR1, peroxisomal hydroxypyruvate reductase; GAP, glyceradehyde-3-phosphate; PGA, 3-phosphoglycerate; Pi, inorganic phosphate; BPGA, 1,3-bis-phosphoglycerate; Mal, malate; OAA, oxaloacetic acid; OH-PYR, hydroxypyruvate.

The photorespiratory pathway is known to be distributed across the chloroplast, mitochondrion, peroxisome and cytosol compartments. We analyzed the role of photorespiration in metabolic readjustments which may occur with changing light conditions. There was increase in flux through photorespiratory pathway with increasing light intensity or photon-flux as can be noted from flux-plot for HPR1/’per_HPR1_RXN’ in **Figure 6A**. This observation supports the view that photorespiratory metabolism may serve to dissipate excess light energy and mitigate photoinhibition (Wu et al., 1991; Heber et al., 1996; Kozaki and Takeba, 1996; Wingler et al., 2000; Voss et al., 2013). At different levels of light the NADH demand of peroxisomal hydroxy pyruvate reductase (HPR1) could be fulfilled in different ways while maintaining cellular energy demand and synthesis of biomass. For this purpose, different combinations of reactions and transporters of the four compartments might be used. Primarily, the chloroplastic TPTs and the dicarboxylate transporters of the chloroplast and the mitochondrion became active for different ranges of light as described below. With increasing photorespiration, mitochondrion import of cytosolic malate declined till it became inactive under medium light ranges. While at high light, MalDH operated to generate malate which was increasingly exported from the mitochondrion (**Figures 6A,B**). With further increase in light, the chloroplastic triose phosphate exchange reactions became active to fulfill the increasing reductant demand of HPR1 (**Figures 6A,C**; the reactions shown in color corresponds to their flux-plot). GAP, formed through PGK and GAPDH catalyzed reactions of the Calvin cycle was exported to the cytosol. In the cytosol, GAP to PGA conversion took place and the PGA thus produced was recycled back to the chloroplast. Chloroplastic reductants and ATP were made available to the cytosol through this recycling between PGA and GAP. This reductant was exported to the peroxisome and made available for HPR1 through Mal-OAA shuttle and malate dehydrogenase catalyzed reactions of the cytosol and peroxisome compartments. Thus, photorespiratory metabolism facilitates export of ATP and reducing equivalents from the chloroplast. Increased light stress leads to generation of reactive oxygen species in photosynthetic systems and photorespiration alleviates this stress especially in the chloroplast (Igamberdiev et al., 2001). Further, the preferred use of alternative oxidase (AOX) component of the mitochondrial ETC over the cytochrome oxidase (COX) component with increasing light, decreased the production, and supply of mitochondrial ATP to cytosol (**Figure 6A**). This is because the AOX component of the ETC decouples ATP generation from redox dissipation (Mackenzie and McIntosh, 1999). As such, ATP demand for cytosolic metabolism was supplied by the GAP to PGA conversion, as discussed above. Previous experimental reports show that the mitochondrial ETC was found to be always active; however, variations in its utilization occurred to optimize photosynthetic performance (Padmasree et al., 2002). We also observed that there was gradual decline of the chloroplastic triose phosphate exchange reactions as the source of reductant (**Figures 6C,D**; the differences in arrow-width represent changes in respective transporter-fluxes). Simultaneously, the fluxes of chloroplastic MalDH (chl_MalDH) and malate valve increased to supply reductant to the cytosol which was finally used in the HPR1 catalyzed reaction (**Figures 6A,D**). The chloroplastic triose phosphate and Mal-OAA transporters are important shuttle systems for transporting reducing equivalents out of the chloroplast. In a study on NADP-MDH Arabidopsis mutants, the NADP-GAPDH was the only alternative that operated to supply reductants to the cytosol (Hebbelmann et al., 2011). While the production of GAP through NADP-GAPDH and PGK catalyzed reactions of the C3 cycle requires both NADPH and ATP, malate production by chl_MalDH requires only NADPH. Thus, in comparison to the triose-phosphate exchange reactions the malate valve acts as a more effective transporter of reductants (Noctor and Foyer, 2000). According to photorespiratory conditions and corresponding ATP/NADPH ratios either of the shuttle systems or both may be used as we have observed. The ATP supplied to the cytosol from the chloroplast decreased with the decline of the chloroplastic triose phosphate exchange reactions and there was a resurgence of mitochondrion complex V which was fuelled by photorespiratory glycine oxidation. Finally, the chloroplastic triose phosphate shuttle became inactive. The activity of the chloroplastic Mal-OAA transporter gradually decreased with concomitant gradual increase in activity of MalDH and Mal-OAA transporters of the mitochondrion and peroxisome compartments (**Figures 6A,E**). Although at low and medium light we found that glycine oxidation was coupled to oxidative phosphorylation (Douce et al., 1991), at high light it was coupled to MalDH (Reumann et al., 1994; Leegood et al., 1995). Thus, we observed interplay of reactions which are parts of different metabolic pathways such as photorespiration, mitochondrion ETC and glycolysis in playing an important role in plant adaptation to increased light-stress. Lastly, the chloroplast and mitochondrial metabolism showed agreement with the observations reported on rice japonica model under light scan (Poolman et al., 2013) and with other previously reported experimental results such as inactivation of pyruvate dehydrogenase in light (Padmasree et al., 2002). Like the previously reported rice japonica model, here light inhibition of pyruvate dehydrogenase was observed although no enzyme kinetics or regulation was included in the simulation.

### Reaction Flux Correlation with *V*_c_/*V*_o_ and Light Intensity

**FIGURE 7:**
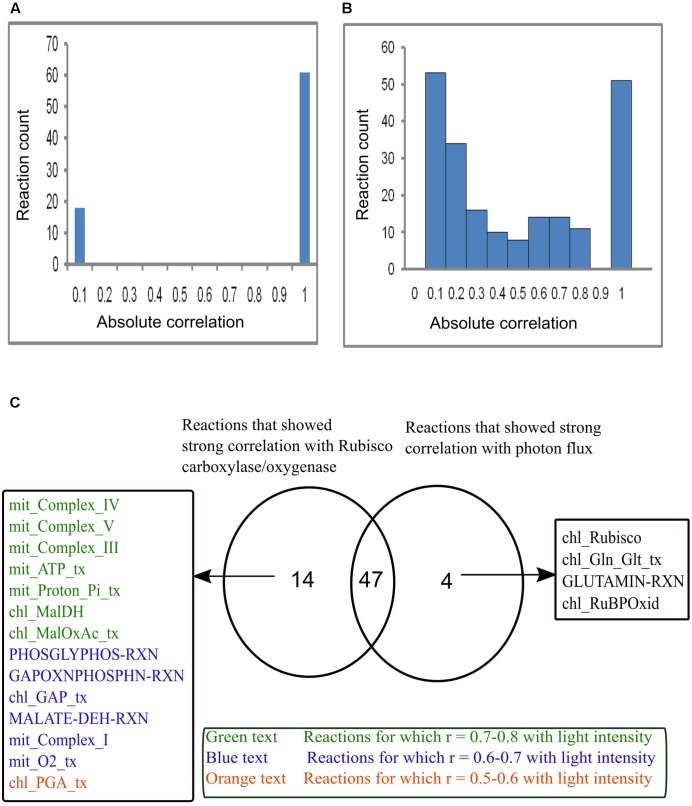
Reaction flux correlation with RuBisCO carboxylase/oxygenase and light intensity. **(A)** Reaction flux correlation with RuBisCO carboxylase/oxygenase. **(B)** Reaction flux correlation with light intensity. These were calculated as the absolute values of the pairwise (Pearson’s) correlation coefficients. The reactions that showed no change in flux were excluded. **(C)** Venn diagram showing comparison of the reactions that showed strong correlation (correlation coefficient, *r* varies between 0.9–1) with RuBisCO carboxylase/oxygenase and light intensity. Forty-seven reactions were found to be strongly correlated with both RuBisCO carboxylase/oxygenase and light intensity. Fourteen reactions that were highly correlated with only RuBisCO carboxylase/oxygenase showed varied correlation with light intensity. Four reactions were highly correlated only with light intensity. chl_ and mit_ represent the reactions present in the chloroplast and mitochondrion respectively. _tx represents the transport reactions.

Reaction correlation with *V*_c_/*V*_o_ and light intensity was calculated and is shown in **Figure 7**. The reactions involved under varying *V*_c_/*V*_o_ were either found to be very weakly or strongly correlated with RuBisCO carboxylase/oxygenase (**Figure 7A**). On the other hand, reaction fluxes correlated with light intensity over the range 0.0–1.0 (**Figure 7B**). **Figure 7C** shows the comparison of reactions that were found to be strongly correlated either with RuBisCO carboxylase/oxygenase or light intensity or both. Forty-seven reactions were found to be strongly correlated with both RuBisCO carboxylase/oxygenase activity and light intensity. This included the reactions of photorespiration in the chloroplast, mitochondrion and peroxisome and the reactions associated with Calvin cycle. A study showed that genes associated with Calvin cycle and photorespiration are co-expressed (Foyer and Noctor, 2011). Also, as discussed before, an equivalent amount of mitochondrial ammonia released into the cytosol was imported into the chloroplast where it was completely utilized in the GS/GOGAT pathway. Accordingly, the mitochondrial and the chloroplastic ammonia transporters and the GS/GOGAT associated reactions showed high correlation with photorespiration. The chloroplastic Mal-2OG and Mal-Glu transporters which were always coupled with the GS/GOGAT pathway (**Figure 6A**) were also found to be highly correlated with RuBisCO carboxylase/oxygenase activity and light intensity. Some of the reactions that showed high correlation only with RuBisCO carboxylase/oxygenase were found to be moderately correlated with light intensity (**Figure 7**). This was because the fluxes of these reactions namely mitochondrial complex V (mit_Complex_V), chloroplastic transporters for Mal-OAA (chl_Maloxac_tx), GAP (chl_GAP_tx), and PGA (chl_PGA_tx) showed significant variations with increase in light intensity **Figure 6**. Cytosolic glutaminase reaction (GLUTAMIN-RXN) and chloroplastic Glu-Gln transport (chl_Gln_Glt_tx) were found to be highly correlated with light intensity but not with *V*_c_/*V*_o_ (**Figure 7C**). With increase in light intensity, flux through cyclic photophosphorylation (chl_LightCyc), and thus ATP generated in the chloroplast increased. Cytosolic glutaminase reaction (GLUTAMIN-RXN) and glutamate-glutamine transporter (chl_Gln_Glt_tx) operated along with chloroplastic glutamine synthetase (GS2) and the ammonia transporter across the chloroplast to utilize the ATP.

### Mimicking the Different Metabolic States

Different enzymatic cost conditions or metabolic states can be mimicked by assigning weights to the reactions and transporters present in a metabolic model (Shaw and Kundu, 2013; Cheung et al., 2015). In a recent work, method of flux weighting factors was implemented to a diel genome-scale model of Arabidopsis leaf metabolism to explore alternative pathways (Cheung et al., 2015). Here, we assigned weights ranging from 0 to 1000 randomly to all the reactions (except the transporters and cyclic and non-cyclic photophosphorylation) and simulated the metabolism for 10000 times under two conditions Case 1 – RuBisCO carboxylase/oxygenase activity (*V*_c_/*V*_o_) was fixed at 3:1 and Case 2 – RuBisCO carboxylase/oxygenase activity was not fixed in any ratio. Plants have acquired several adaptive mechanisms including flexibility in metabolic pathways to combat different environmental stress conditions (Bohnert et al., 1995). Accordingly, the simulations for mimicking varying enzymatic costs show existence of different modes of activity of reactions and transporters (discussed below).

### Variation in the Participation of Glutathione-Ascorbate Cycle

It is known that ascorbate and glutathione lie at the heart of the redox hub to combat oxidative stress (Foyer and Noctor, 2011) and as photorespiration is a major source of hydrogen-peroxide in peroxisomes, the involvement of glutathione-ascorbate cycle in dissipating it was analyzed (Queval et al., 2007). With *V*_c_/*V*_o_ fixed at 3:1, it was found that peroxisomal glutathione-ascorbate cycle was used instead of catalase to metabolize photorespiratory hydrogen-peroxide 116 times out of 10000 simulations. Further, we examined its participation under different ranges of light, based on our light scan analysis. For this the photon flux was set at different ranges. Under low light glutathione-ascorbate cycle operated as an alternative to catalase only about 1% of the times the number of solution spaces that were obtained for the simulations. Under medium and high light conditions it operated 16% of the times. Also, about 0.5% of the times under medium and high light conditions the glutathione-ascorbate cycle operated together with catalase. Such a case of concomitant operation of glutathione-ascorbate cycle and catalase was not observed for low light conditions. Although the above analysis was carried out over a range of photon flux, participation of glutathione-ascorbate cycle was absent from the solutions obtained for varying light intensity. Thus, the participation of glutathione-ascorbate cycle was independent of light intensity and reflective of the different enzymatic cost considered.

### Chloroplastic Triose Phosphate Transporters and the Malate Valve

Previous studies suggested that plants can activate different metabolic reactions and transporters depending on their cellular need (Poolman et al., 2009; Sweetlove et al., 2010). In the present study, we simulated the metabolic model for different weights assigned to the reactions in the model (see Materials and Methods for details). For each simulation, a feasible solution was obtained. Each solution consisted of metabolic reactions and transporters which became operative under the simulated conditions. It may happen that under a certain condition a particular reaction or a transporter is active, while under another condition it gets replaced by some other reaction or a transporter. Another possibility can be the change in the direction in which a reaction or a transporter is active (if it is reversible). In our results, if any of these changes occurred between the feasible solutions, then they were termed as different modes.

When *V*_c_/*V*_o_ was fixed at 3:1, 236 modes were found which showed differences in the activity of the chloroplastic and the mitochondrial transporters. The chloroplastic TPTs and the malate valve showed considerable variation in their activity under different enzymatic cost conditions. The TPTs can operate in either direction depending on the availability of the redox equivalents (NAD or NADP) (Heber and Heldt, 1981). Most of the modes include the export of dihydroxy acetone phosphate (DHAP) and GAP from the chloroplast to the cytosol and import of PGA from the cytosol to the chloroplast along with exchange of phosphate (Pi). This activity of TP/3PGA shuttle is well described in plants (Flügge and Heldt, 1984). The results further indicated that at different cellular enzymatic costs plants may prefer the above-mentioned mode of activity (**Figure 3**). Variation in the activity of the enzymes of malate valve is known to occur in plants under different conditions like high light, high CO_2_, cold stress etc (Scheibe, 2004). Our results also showed that under different enzymatic cost conditions (that is expected at different stress/which corresponds to different conditions to which plants adapt) the malate valve operated differently. Most of the times (111/236) the Mal-OAA shuttle operated to import OAA into the chloroplast, followed by reduction of OAA to Mal and then the export of Mal out of the chloroplast (**Figure 3**). This activity of the malate valve which acts as an indirect export system for reducing equivalents serves to balance the ATP/NADPH ratio in the chloroplast (Backhausen et al., 1998). Fifty-seven modes showed inactivity of the malate valve. Inactivity of NADP–MDH is known to occur under conditions when the NADP in the chloroplast reached high levels preventing export of reducing equivalents as malate (Scheibe, 1990). The results also showed that 68 times the malate valve operated in reverse manner.

### Different Modes of Chloroplastic Glu–Gln Transporter and Its Relation with the Activity of GS1 and GS2

**FIGURE 8:**
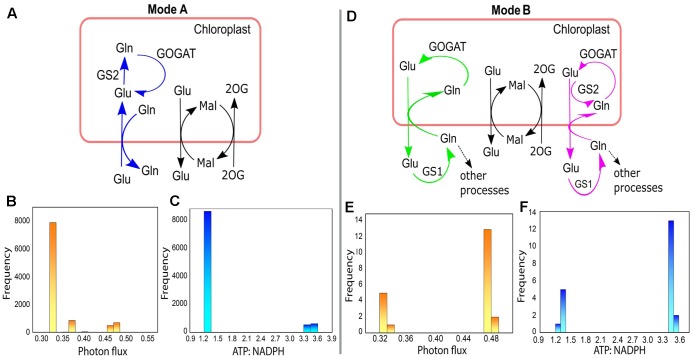
Different modes of chloroplastic Glu–Gln transporter under varying enzymatic costs. **(A)** Mode A – chloroplastic Glu–Gln transporter operated to import Glu into the chloroplast and export Gln out of the chloroplast. GS2 was operative but not GS1. **(B)** Frequency plot showing range of photon flux for Mode A. **(C)** Frequency plot showing ATP:NADPH ratio for Mode A. **(D)** Mode B – chloroplastic Glu–Gln transporter operated to export Glu out of the chloroplast and import Gln into the chloroplast. Here, GS2 was not operative (marked in green) except for once when both GS1 and GS2 were active (marked in pink). The transporters for Mal-2OG and Mal-Glu were always operative with the same flux. **(E)** Frequency plot showing range of photon flux for Mode B. **(F)** Frequency plot showing ATP:NADPH ratio for Mode B. Glu, glutamate; Gln, glutamine; Mal, malate; 2OG, 2ketoglutarate; GS1, cytosolic glutamine synthetase; GS2, chloroplastic glutamine synthetase; GOGAT, glutamate synthase.

For the 10000 optimal solutions obtained under different enzymatic cost conditions (with or without *V*_c_/*V*_o_ fixed at 3:1) we found that different modes of chloroplastic Glu–Gln transporter operated. It operated maximum number of times (9978/10000; Mode A) to import Glu into the chloroplast and export Gln out of the chloroplast (**Figure 8A**). At other times (21/10000; Mode B) it operated in reverse direction, i.e., Glu was exported out of the chloroplast and Gln entered into the chloroplast (**Figure 8D**) except once where it was found to be inactive. In, Mode A, most of the times low photon flux was required (≈ 0.33–0.33 flux units) and in Mode B the photon flux was in the range of 0.47–0.48 flux units (**Figures 8B,E**, respectively). Concomitantly, the ATP:NADPH ratio was less for Mode A than for Mode B (**Figures 8C,F**, respectively). These observations were related to changes in the cytosolic (GS1) and chloroplastic (GS2) glutamine synthetase activities. The relative amounts of these isoenzymes GS1 and GS2 (Kamachi et al., 1992) are known to change with leaf development (Yamaya et al., 1992). Further, a study on tobacco reported that under high photosynthetic capacity GS2 was more active but during leaf aging, expression of GS1 increased (Masclaux et al., 2000). In Mode A, GS2 was operative while GS1 was inactive. On the other hand, in Mode B, GS1 was operative and GS2 was inactive except for once when both operated simultaneously (**Figure 8D**). GS2 is the predominant isoform of GS in C3 species (McNally et al., 1983) and photorespiratory ammonia is refixed by GS2 (Keys, 2006). This role of GS2 was also illustrated from the results of FBA that we described in **Figure 5B**. Further, the occurrence of both GS1 and GS2, as described in Mode B, was in agreement with the study which had showed involvement of both cytosolic and chloroplastic GS isoforms in ammonia assimilation (Yu and Woo, 1988). Reaction catalyzed by GOGAT was active throughout to supply Glu which acts as a precursor for chlorophyll synthesis (Chatterjee and Kundu, 2015) and is known to occupy the central position in photorespiratory nitrogen metabolism (Masclaux-Daubresse et al., 2006). Altogether, the results of varying enzymatic conditions implied that the chloroplastic Glu–Gln transporter may show different modes of activity related to the functioning of GS1 and GS2 and that GS1 may serve as an alternative route for stress adaptation.

## Conclusion

In this work, we reconstructed a GSM for *O.s. indica* and studied its responses to various conditions using FBA. The results allow us to conclude the following:

(1) The increased activity of the malate valve under drought stress indicates its possible role in transporting reducing equivalents out of the chloroplast.
(2) GAPDH and PGK can also act as source of cytosolic ATP when *V*_c_/*V*_o_ increases (i.e., under decreased photorespiration).
(3) For the light conditions investigated, we have presented a coherent mechanism of photorespiratory metabolism in conjunction with other reactions namely mitochondrion ETC, glycolysis, and intracellular transporters which function to maintain cellular ATP and NAD(P)H homeostasis.
(4) Under different enzymatic cost conditions there exists variation in participation of peroxisomal glutathione-ascorbate cycle and different modes of the chloroplastic TPTs and the malate valve may become operative. Also, chloroplastic Glu–Gln transporter may show different modes of activity related with the functioning of GS1 and GS2, which may serve as alternative routes for stress adaptation in plants.

## Author Contributions

AC and BH reconstructed the model. AC, BH, and RS carried out simulation and analysis. AC and BH wrote the manuscript. SK supervised the work.

## Conflict of Interest Statement

The authors declare that the research was conducted in the absence of any commercial or financial relationships that could be construed as a potential conflict of interest.

## Footnotes

1 http://www.gnu.org/software/glpk/
