# Supplementary material for: Reconstruction of Oryza sativa indica Genome Scale Metabolic Model and Its Responses to Varying RuBisCO Activity, Light Intensity, and Enzymatic Cost Conditions
Source: Front Plant Sci. 2017 Nov 30;8:2060. doi: 10.3389/fpls.2017.02060 (PMC5715477; doi:10.3389/fpls.2017.02060)
Supplement: Supplementary file 1 [file Table_1.DOC]

**Table S1.** List of reactions removed from the model. 206 reactions were removed from the draft model.

| **Reaction Name** | **Reaction** |
| --- | --- |
| **##19 reactions with acceptors** |  |
| RXN-821 | 1/1 PRO + 1/1 Acceptor <> 1/1 L-DELTA1-PYRROLINE_5-CARBOXYLATE + 1/1 Donor-H2 |
| 2.8.1.6-RXN | 1/1 Sulfurated-Sulfur-Acceptors + 1/1 DETHIOBIOTIN + 2/1 S-ADENOSYLMETHIONINE -> 1/1 BIOTIN + 2/1 CH33ADO + 2/1 MET + 1/1 Unsulfurated-Sulfur-Acceptors |
| 3.2.1.21-RXN | 1/1 Glucosylated-Glucose-Acceptors + 1/1 WATER -> 1/1 Non-Glucosylated-Glucose-Acceptors + 1/1 GLC |
| 3.2.1.23-RXN | 1/1 Galactosylated-Galactose-Acceptors + 1/1 WATER -> 1/1 Non-Galactosylated-Galactose-Acceptors + 1/1 GALACTOSE |
| 1.14.99.30-RXN | 1/1 NEUROSPORENE + 1/1 OXYGEN-MOLECULE + 1/1 Donor-H2 -> 1/1 CPD1F-114 + 2/1 WATER + 1/1 Acceptor |
| RXN-10666 | 1/1 CPD-11463 + 1/1 OXYGEN-MOLECULE + 1/1 Donor-H2 -> 1/1 CPD-11464 + 2/1 WATER + 1/1 Acceptor |
| RXN-10667 | 1/1 CPD-11469 + 1/1 OXYGEN-MOLECULE + 1/1 Donor-H2 -> 1/1 CPD-11468 + 2/1 WATER + 1/1 Acceptor |
| RXN-8022 | 1/1 CPD1F-98 + 1/1 OXYGEN-MOLECULE + 1/1 Donor-H2 -> 1/1 NEUROSPORENE + 2/1 WATER + 1/1 Acceptor |
| SQUALENE-MONOOXYGENASE-RXN | 1/1 SQUALENE + 1/1 OXYGEN-MOLECULE + 1/1 Donor-H2 -> 1/1 EPOXYSQUALENE + 1/1 WATER + 1/1 Acceptor |
| HEME-OXYGENASE-DECYCLIZING-RXN | 1/1 PROTOHEME + 3/1 OXYGEN-MOLECULE + 3/1 Donor-H2 -> 1/1 BILIVERDINE + 1/1 FE+2 + 1/1 CARBON-MONOXIDE + 3/1 Acceptor + 3/1 WATER |
| RXN0-2301 | 1/1 ISOVALERYL-COA + 1/1 Acceptor -> 1/1 3-METHYL-CROTONYL-COA + 1/1 Donor-H2 |
| 1.14.19.2-RXN | 1/1 Stearoyl-ACPs + 1/1 Donor-H2 + 1/1 OXYGEN-MOLECULE -> 1/1 Oleoyl-ACPs + 1/1 Acceptor + 2/1 WATER |
| DEOXYHYPUSINE-MONOOXYGENASE-RXN | 1/1 CPD-9973 + 1/1 OXYGEN-MOLECULE + 1/1 Donor-H2 -> 1/1 EIF5A-HYPUSINE + 1/1 WATER + 1/1 Acceptor |
| KYNURENINE-78-HYDROXYLASE-RXN | 1/1 OXYGEN-MOLECULE + 1/1 Donor-H2 + 1/1 KYNURENATE -> 1/1 Acceptor + 1/1 CPD-274 |
| SUCC-FUM-OXRED-RXN | 1/1 SUC + 1/1 Acceptor <> 1/1 FUM + 1/1 Donor-H2 |
| 1.5.99.12-RXN | 1/1 CPD-4209 + 1/1 Acceptor + 1/1 WATER -> 1/1 PRENAL + 1/1 Donor-H2 + 1/1 ADENINE |
| SARCOSINE-DEHYDROGENASE-RXN | 1/1 WATER + 1/1 Acceptor + 1/1 SARCOSINE -> 1/1 Donor-H2 + 1/1 FORMALDEHYDE + 1/1 GLY |
| NADH-DEHYDROGENASE-RXN | 1/1 Acceptor + 1/1 NADH -> 1/1 Donor-H2 + 1/1 NAD |
| 1.6.99.5-RXN | 1/1 NADH + 1/1 PROTON + 1/1 Acceptor -> 1/1 NAD + 1/1 Donor-H2 |
| **##10 reactions with peptides** |  |
| 3.4.19.9-RXN | 1/1 Protein-gamma-glutamates -> 1/1 GLT + 1/1 Peptides |
| 3.4.25.1-RXN | 1/1 General-Protein-Substrates -> 1/1 Peptides + 1/1 Peptides |
| 3.4.21.92-RXN | 1/1 General-Protein-Substrates -> 1/1 Peptides + 1/1 Peptides |
| 3.4.21.89-RXN | 1/1 Peptides-with-Leader-Sequence -> 1/1 Peptides + 1/1 Leader-Sequences |
| ACYLAMINOACYL-PEPTIDASE-RXN | 1/1 N-Acetyl-Peptides + 1/1 WATER -> 1/1 Peptides + 1/1 N-Acylated-Amino-Acids |
| GAMMA-GLUTAMYLTRANSFERASE-RXN | 1/1 5-L-GLUTAMYL-PEPTIDE + 1/1 Amino-Acids-20 -> 1/1 5-L-GLUTAMYL-L-AMINO-ACID + 1/1 Peptides |
| 2.3.1.97-RXN | 1/1 GLYCYL-PEPTIDE + 1/1 TETRADECANOYL-COA -> 1/1 N-TETRADECANOYLGLYCYL-PEPTIDE + 1/1 CO-A |
| PEPTIDE-ALPHA-N-ACETYLTRANSFERASE-RXN | 1/1 ACETYL-COA + 1/1 Peptides -> 1/1 N-ACETYLPEPTIDE + 1/1 CO-A |
| 3.4.22.1-RXN | 1/1 General-Protein-Substrates -> 1/1 Peptides + 1/1 Peptides |
| 3.4.11.1-RXN | 1/1 Peptides + 1/1 WATER -> 1/1 Amino-Acids-20 + 1/1 Peptides |
| **##50 incomplete reactions** |  |
| 3.4.11.10-RXN |  |
| 3.4.11.18-RXN |  |
| 3.4.11.5-RXN |  |
| 3.4.13.9-RXN |  |
| 3.4.16.5-RXN |  |
| 3.4.16.6-RXN |  |
| 3.4.19.5-RXN |  |
| 3.4.21.53-RXN |  |
| 3.4.21.9-RXN |  |
| 3.5.1.52-RXN |  |
| 5.99.1.2-RXN |  |
| 5.99.1.3-RXN |  |
| 3.2.1.11-RXN |  |
| 3.2.1.113-RXN |  |
| 3.4.22.16-RXN |  |
| 3.2.1.37-RXN |  |
| 3.2.1.55-RXN |  |
| 3.4.21.1-RXN |  |
| 3.4.21.10-RXN |  |
| 3.2.2.21-RXN |  |
| 3.4.11.9-RXN |  |
| 3.4.24.6-RXN |  |
| 3.4.24.64-RXN |  |
| 3.2.1.39-RXN |  |
| 3.4.11.21-RXN |  |
| 3.4.22.3-RXN |  |
| 3.4.22.34-RXN |  |
| 3.4.24.40-RXN |  |
| 3.2.2.23-RXN |  |
| 3.4.21.2-RXN |  |
| 3.4.21.26-RXN |  |
| 3.4.23.4-RXN |  |
| 3.4.23.40-RXN |  |
| 2.5.1.5-RXN |  |
| 3.1.11.5-RXN |  |
| RXN0-2605 |  |
| 3.1.21.7-RXN |  |
| 3.1.26.1-RXN |  |
| 3.1.26.11-RXN |  |
| 3.1.26.4-RXN |  |
| 3.1.27.1-RXN |  |
| 3.1.27.9-RXN |  |
| 3.1.4.1-RXN |  |
| 3.2.1.14-RXN |  |
| RXN0-4082 |  |
| 3.2.1.7-RXN |  |
| 3.2.1.78-RXN |  |
| 3.2.1.6-RXN |  |
| 2.4.1.207-RXN |  |
| 2.4.1.24-RXN |  |
| **#119 reactions that are non-plant/involve substrates or products like RNA,DNA,Folate,ssRNA,mRNA,tRNA/similar** |  |
| **DEXTRIN-DEXTRANASE-RXN** | 1/1 1-4-alpha-D-Glucan+ 1/1 1-6-alpha-D-glucan-> 1/1 1-4-alpha-D-Glucan+ 1/1 1-6-alpha-D-glucan |
| 3.2.1.68-RXN | 1/1 1-6-alpha-D-glucan+ 1/1 WATER-> 1/1 1-6-alpha-D-glucan+ 1/1 MALTOSE |
| DNA-DIRECTED-RNA-POLYMERASE-RXN | 1/1 Nucleoside-Triphosphates+ 1/1 RNAs-> 1/1 PPI+ 1/1 RNAs |
| RNA-DIRECTED-DNA-POLYMERASE-RXN | 1/1 Deoxy-Ribonucleoside-Triphosphates+ 1/1 DNA-N-> 1/1 PPI+ 1/1 DNA-N |
| DNA-LIGASE-ATP-RXN | 1/1 ATP+ 1/1 Deoxynucleotides+ 1/1 DEOXYNUCLEOTIDESM-> 1/1 AMP+ 1/1 PPI+ 1/1 Deoxynucleotides |
| RNA-DIRECTED-RNA-POLYMERASE-RXN | 1/1 RNAs+ 1/1 Nucleoside-Triphosphates-> 1/1 RNAs+ 1/1 PPI |
| RXN-7668 | 1/1 UDP-GLUCOSE+ 1/1 POLY-GLUCOSYLATED-GLYCOGENINS-> 1/1 POLY-GLUCOSYLATED-GLYCOGENINS+ 1/1 UDP |
| RXN-3741 | 1/1 All-Folates+ 1/1 WATER-> 1/1 GLT+ 1/1 All-Folates |
| ALCOHOL-DEHYDROG-GENERIC-RXN | 1/1 Alcohols+ 1/1 NAD<> 1/1 Aldehydes-Or-Ketones+ 1/1 NADH+ 1/1 PROTON |
| RXN-9597 | 1/1 Primary-Amines+ 1/1 WATER+ 1/1 OXYGEN-MOLECULE-> 1/1 Aldehydes+ 1/1 AMMONIA+ 1/1 HYDROGEN-PEROXIDE |
| RXN-8635 | 2/1 CPD-8653+ 1/1 HYDROGEN-PEROXIDE-> 1/1 CPD-8653+ 1/1 CPD-8890+ 2/1 WATER |
| 2.4.1.242-RXN | 1/1 NDP-glucoses+ 1/1 1-4-alpha-D-Glucan-> 1/1 Nucleoside-Diphosphates+ 1/1 1-4-alpha-D-Glucan |
| 3.4.11.2-RXN | 1/1 POLYPEPTIDE-> 2/1 POLYPEPTIDE |
| AMYLOSUCRASE-RXN | 1/1 SUCROSE+ 1/1 1-4-alpha-D-Glucan-> 1/1 FRU+ 1/1 1-4-alpha-D-Glucan |
| L-AMINO-ACID-OXIDASE-RXN | 1/1 OXYGEN-MOLECULE+ 1/1 WATER+ 1/1 Amino-Acids-20-> 1/1 AMMONIA+ 1/1 HYDROGEN-PEROXIDE+ 1/1 2-Oxo-Acids |
| RXN-6601 | 1/1 5-L-GLUTAMYL-L-AMINO-ACID+ 1/1 CYS-GLY<- 1/1 Amino-Acids-20+ 1/1 GLUTATHIONE |
| RXN-9917 | 1/1 Long-Chain-Fatty-Acids+ 1/1 CO-A+ 1/1 ATP-> 1/1 Fatty-Acyl-CoA+ 1/1 PPI+ 1/1 AMP |
| BIOTIN-CARBOXYL-RXN | 1/1 BCCP-dimers+ 1/1 HCO3+ 1/1 ATP<> 1/1 Pi+ 1/1 Carboxybiotin-BCCP+ 1/1 ADP |
| 2.7.7.8-RXN | 1/1 RNAs+ 1/1 Pi<> 1/1 RNAs+ 1/1 Nucleoside-Diphosphates |
| PSERPHOSPHA-RXN | 1/1 Phosphoserines+ 1/1 WATER-> 1/1 Serines+ 1/1 Pi |
| RXN-2103 | 1/1 1-4-alpha-D-galacturonosyl+ 1/1 WATER-> 1/1 1-4-alpha-D-galacturonosyl+ 1/1 1-4-alpha-D-galacturonosyl |
| RXN-11332 | 1/1 Primary-Alcohols+ 2/1 Cytochromes-CL-Ox-> 1/1 Aldehydes+ 2/1 Cytochromes-CL-Red |
| RXN-11034 | 1/1 CPD-12004+ 1/1 NAD+<> 1/1 CPD-255+ 1/1 NADH+ 1/1 PROTON |
| RXN-11035 | 1/1 CPD-12004+ 1/1 NAD<> 1/1 CPD-10353+ 1/1 NADH+ 1/1 PROTON |
| RXN-8642 | 2/1 PROTON+ 1/1 OXALO-SUCCINATE+ 1/1 NADP-> 1/1 2-KETOGLUTARATE+ 1/1 CARBON-DIOXIDE+ 1/1 NADPH |
| CATECHOL-12-DIOXYGENASE-RXN | 1/1 CATECHOL+ 1/1 OXYGEN-MOLECULE-> 2/1 PROTON+ 1/1 CIS-CIS-MUCONATE |
| CATECHOL-2-3-DIOXYGENASE-RXN | 1/1 CPD-111+ 1/1 OXYGEN-MOLECULE-> 1/1 PROTON+ 1/1 2H6OH2-4DIENOATE |
| CATECHOL-23-DIOXYGENASE-RXN | 1/1 CATECHOL+ 1/1 OXYGEN-MOLECULE<> 1/1 PROTON+ 1/1 HYDROXYMUCONATE-SALD |
| RXN-11218 | 1/1 CPD-111+ 1/1 OXYGEN-MOLECULE-> 1/1 CPD-8782+ 1/1 PROTON |
| RXN-11219 | 1/1 4-METHYLCATECHOL+ 1/1 OXYGEN-MOLECULE-> 1/1 CPD-8781+ 1/1 PROTON |
| PHENOL-2-MONOOXYGENASE-RXN | 1/1 PROTON+ 1/1 OXYGEN-MOLECULE+ 1/1 NADPH+ 1/1 PHENOL-> 1/1 WATER+ 1/1 NADP+ 1/1 CATECHOL |
| 2.4.1.11-RXN | 1/1 UDP-GLUCOSE+ 1/1 1-4-alpha-D-Glucan-> 1/1 1-4-alpha-D-Glucan+ 1/1 UDP |
| POLYNUCLEOTIDE-ADENYLYLTRANSFERASE-RXN | 1/1 ATP+ 1/1 mRNAs-> 1/1 PPI+ 1/1 mRNAs |
| DNA-DIRECTED-DNA-POLYMERASE-RXN | 1/1 Deoxy-Ribonucleoside-Triphosphates+ 1/1 DNA-N-> 1/1 PPI+ 1/1 DNA-N |
| RXN0-1483 | 4/1 FE+2+ 4/1 PROTON+ 1/1 OXYGEN-MOLECULE-> 4/1 FE+3+ 2/1 WATER |
| 3.1.26.5-RXN | 1/1 CPD0-2352-> 1/1 CPD0-2353+ 1/1 ssRNAs |
| RXN0-6480 | 1/1 CPD0-2354-> 1/1 tRNAs+ 1/1 ssRNAs |
| RXN0-6528 | 1/1 YHAV-DEGRADATION-SUBSTRATE-MRNA-> 2/1 ssRNAs |
| RXN0-6529 | 1/1 YHAV-DEGRADATION-SUBSTRATE-RRNA-> 2/1 ssRNAs |
| 3.1.26.3-RXN | 1/1 RNASE-III-MRNA-PROCESSING-SUBSTRATE-> 1/1 RNASE-III-PROCESSING-PRODUCT-MRNA+ 2/1 ssRNAs |
| AMINOCYL-TRNA-HYDROLASE-RXN | 1/1 WATER+ 1/1 N-Substituted-Aminoacyl-tRNA-> 1/1 All-tRNAs+ 1/1 N-Substituted-Amino-Acids |
| MUCONATE-CYCLOISOMERASE-RXN | 1/1 CPD-741<- 1/1 PROTON+ 1/1 CIS-CIS-MUCONATE |
| 1.1.1.39-RXN | 1/1 MAL+ 1/1 NAD-> 1/1 NADH+ 1/1 CARBON-DIOXIDE+ 1/1 PYRUVATE |
| TYROSINE--TRNA-LIGASE-RXN | 1/1 PROTON+ 1/1 TYR-tRNAs+ 1/1 TYR+ 1/1 ATP-> 1/1 Charged-TYR-tRNAs+ 1/1 PPI+ 1/1 AMP |
| METHIONINE--TRNA-LIGASE-RXN | 1/1 PROTON+ 1/1 MET-tRNAs+ 1/1 MET+ 1/1 ATP-> 1/1 Charged-MET-tRNAs+ 1/1 PPI+ 1/1 AMP |
| RXN0-2161 | 1/1 PROTON+ 1/1 SEC-tRNAs+ 1/1 SER+ 1/1 ATP-> 1/1 L-seryl-SEC-tRNAs+ 1/1 PPI+ 1/1 AMP |
| SERINE--TRNA-LIGASE-RXN | 1/1 PROTON+ 1/1 SER-tRNAs+ 1/1 SER+ 1/1 ATP-> 1/1 Charged-SER-tRNAs+ 1/1 PPI+ 1/1 AMP |
| ASPARTATE--TRNA-LIGASE-RXN | 1/1 PROTON+ 1/1 ASP-tRNAs+ 1/1 L-ASPARTATE+ 1/1 ATP-> 1/1 Charged-ASP-tRNAs+ 1/1 PPI+ 1/1 AMP |
| GLYCINE--TRNA-LIGASE-RXN | 1/1 PROTON+ 1/1 GLY-tRNAs+ 1/1 GLY+ 1/1 ATP-> 1/1 Charged-GLY-tRNAs+ 1/1 PPI+ 1/1 AMP |
| PROLINE--TRNA-LIGASE-RXN | 1/1 PROTON+ 1/1 PRO-tRNAs+ 1/1 PRO+ 1/1 ATP-> 1/1 Charged-PRO-tRNAs+ 1/1 PPI+ 1/1 AMP |
| CYSTEINE--TRNA-LIGASE-RXN | 1/1 PROTON+ 1/1 CYS-tRNAs+ 1/1 CYS+ 1/1 ATP-> 1/1 Charged-CYS-tRNAs+ 1/1 PPI+ 1/1 AMP |
| GLUTAMINE--TRNA-LIGASE-RXN | 1/1 PROTON+ 1/1 GLN-tRNAs+ 1/1 GLN+ 1/1 ATP-> 1/1 Charged-GLN-tRNAs+ 1/1 PPI+ 1/1 AMP |
| ARGININE--TRNA-LIGASE-RXN | 1/1 PROTON+ 1/1 ARG-tRNAs+ 1/1 ARG+ 1/1 ATP-> 1/1 Charged-ARG-tRNAs+ 1/1 PPI+ 1/1 AMP |
| TRYPTOPHAN--TRNA-LIGASE-RXN | 1/1 PROTON+ 1/1 TRP+ 1/1 TRP-tRNAs+ 1/1 ATP-> 1/1 AMP+ 1/1 PPI+ 1/1 Charged-TRP-tRNAs |
| PHENYLALANINE--TRNA-LIGASE-RXN | 1/1 PROTON+ 1/1 PHE-tRNAs+ 1/1 PHE+ 1/1 ATP-> 1/1 Charged-PHE-tRNAs+ 1/1 PPI+ 1/1 AMP |
| HISTIDINE--TRNA-LIGASE-RXN | 1/1 PROTON+ 1/1 HIS-tRNAs+ 1/1 HIS+ 1/1 ATP-> 1/1 Charged-HIS-tRNAs+ 1/1 PPI+ 1/1 AMP |
| ASPARAGINE--TRNA-LIGASE-RXN | 1/1 PROTON+ 1/1 ASN-tRNAs+ 1/1 ASN+ 1/1 ATP-> 1/1 Charged-ASN-tRNAs+ 1/1 PPI+ 1/1 AMP |
| THREONINE--TRNA-LIGASE-RXN | 1/1 PROTON+ 1/1 THR-tRNAs+ 1/1 THR+ 1/1 ATP-> 1/1 Charged-THR-tRNAs+ 1/1 PPI+ 1/1 AMP |
| LEUCINE--TRNA-LIGASE-RXN | 1/1 PROTON+ 1/1 LEU-tRNAs+ 1/1 LEU+ 1/1 ATP-> 1/1 Charged-LEU-tRNAs+ 1/1 PPI+ 1/1 AMP |
| ISOLEUCINE--TRNA-LIGASE-RXN | 1/1 PROTON+ 1/1 ILE-tRNAs+ 1/1 ILE+ 1/1 ATP-> 1/1 Charged-ILE-tRNAs+ 1/1 PPI+ 1/1 AMP |
| LYSINE--TRNA-LIGASE-RXN | 1/1 PROTON+ 1/1 LYS+ 1/1 LYS-tRNAs+ 1/1 ATP-> 1/1 Charged-LYS-tRNAs+ 1/1 PPI+ 1/1 AMP |
| ALANINE--TRNA-LIGASE-RXN | 1/1 PROTON+ 1/1 ALA-tRNAs+ 1/1 L-ALPHA-ALANINE+ 1/1 ATP-> 1/1 Charged-ALA-tRNAs+ 1/1 PPI+ 1/1 AMP |
| VALINE--TRNA-LIGASE-RXN | 1/1 PROTON+ 1/1 VAL-tRNAs+ 1/1 VAL+ 1/1 ATP-> 1/1 Charged-VAL-tRNAs+ 1/1 PPI+ 1/1 AMP |
| 2.7.11.22-RXN | 1/1 ATP+ 1/1 General-Protein-Substrates-> 1/1 ADP+ 1/1 General-Phos-Protein-Substrates |
| 2.7.11.24-RXN | 1/1 ATP+ 1/1 General-Protein-Substrates-> 1/1 ADP+ 1/1 General-Phos-Protein-Substrates |
| 2.7.12.1-RXN | 1/1 ATP+ 1/1 General-Protein-Substrates-> 1/1 ADP+ 1/1 General-Phos-Protein-Substrates |
| 2.7.12.2-RXN | 1/1 ATP+ 1/1 General-Protein-Substrates-> 1/1 ADP+ 1/1 General-Phos-Protein-Substrates |
| RNA-POLYMERASE-SUBUNIT-KINASE-RXN | 1/1 ATP+ 1/1 DNA-directed-RNA-polymerases-> 1/1 ADP+ 1/1 Phospho-DNA-directed-RNA-polymerases |
| RXN0-3962 | 1/1 ACETALD+ 1/1 NADP+ 1/1 WATER-> 1/1 ACET+ 1/1 NADPH+ 2/1 PROTON |
| ALANINE-DEHYDROGENASE-RXN | 1/1 NAD+ 1/1 WATER+ 1/1 L-ALPHA-ALANINE<> 2/1 PROTON+ 1/1 AMMONIA+ 1/1 NADH+ 1/1 PYRUVATE |
| RXN-1825 | 1/1 Long-linear-glucans+ 1/1 WATER-> 1/1 Short-glucans |
| DLACTDEHYDROGFAD-RXN | 1/1 D-LACTATE+ 1/1 Quinones-> 1/1 PYRUVATE+ 1/1 Reduced-Quinones |
| RXN-11845 | 1/1 23S-rRNA-uridine-2552+ 1/1 S-ADENOSYLMETHIONINE-> 1/1 23S-rRNA-2-O-methyluridine2552+ 1/1 ADENOSYL-HOMO-CYS |
| RXN-11633 | 1/1 16S-rRNA-adenine1518-adenine1519+ 4/1 S-ADENOSYLMETHIONINE-> 1/1 16S-rRNA-N6-dimethyladenine1518-1519+ 4/1 ADENOSYL-HOMO-CYS |
| RXN-11586 | 1/1 S-ADENOSYLMETHIONINE+ 1/1 23S-rRNA-adenine-2503-> 1/1 ADENOSYL-HOMO-CYS+ 1/1 23S-rRNA-2-methyladenine2503 |
| TRNA-GUANINE-N7--METHYLTRANSFERASE-RXN | 1/1 S-ADENOSYLMETHIONINE+ 1/1 All-tRNAs-> 1/1 ADENOSYL-HOMO-CYS+ 1/1 tRNAs-with-N7-methyl-guanine |
| TRNA-ADENINE-N1--METHYLTRANSFERASE-RXN | 1/1 All-tRNAs+ 1/1 S-ADENOSYLMETHIONINE-> 1/1 ADENOSYL-HOMO-CYS+ 1/1 tRNA-Containing-N1-Methyladenine |
| TRNA-GUANINE-N2--METHYLTRANSFERASE-RXN | 1/1 All-tRNAs+ 1/1 S-ADENOSYLMETHIONINE-> 1/1 ADENOSYL-HOMO-CYS+ 1/1 tRNA-Containing-N2-Methylguanine |
| RXN-11578 | 1/1 S-ADENOSYLMETHIONINE+ 1/1 16S-rRNA-guanine-527-> 1/1 ADENOSYL-HOMO-CYS+ 1/1 16S-rRNA-N7-methylguanine527 |
| MRNA-GUANINE-N7--METHYLTRANSFERASE-RXN | 1/1 G5-pppR-mRNAs+ 1/1 S-ADENOSYLMETHIONINE-> 1/1 ADENOSYL-HOMO-CYS+ 1/1 m7G5-pppR-mRNAs |
| RXN-11373 | 1/1 3-carboxy-3-dimethylammonio-propyl-L-his+ 1/1 S-ADENOSYLMETHIONINE-> 1/1 DIPHTINE+ 1/1 ADENOSYL-HOMO-CYS+ 1/1 PROTON |
| RXN-11374 | 1/1 2-3-CARBOXY-3-METHYLAMMONIOPROPYL-L-+ 1/1 S-ADENOSYLMETHIONINE-> 1/1 3-carboxy-3-dimethylammonio-propyl-L-his+ 1/1 ADENOSYL-HOMO-CYS+ 1/1 PROTON |
| TRNA-PSEUDOURIDINE-SYNTHASE-I-RXN | 1/1 tRNA-uridine-38-40-> 1/1 tRNA-pseudouridine-38-40 |
| RXN0-6515 | 1/1 S-ADENOSYLMETHIONINE+ 1/1 16S-rRNA-guanine-966-> 1/1 ADENOSYL-HOMO-CYS+ 1/1 16S-rRNA-N2-methylguanine966 |
| RXN-11596 | 1/1 23S-rRNA-adenine-1618+ 1/1 S-ADENOSYLMETHIONINE-> 1/1 23S-rRNA-N6-methyladenine1618+ 1/1 ADENOSYL-HOMO-CYS |
| RXN-11634 | 1/1 18S-rRNA-adenine1779-adenine1780+ 4/1 S-ADENOSYLMETHIONINE-> 1/1 18S-rRNA-N6-dimethyladenine1779-1780+ 4/1 ADENOSYL-HOMO-CYS |
| ADENYLATECYC-RXN | 1/1 ATP-> 1/1 CAMP+ 1/1 PPI |
| TRANSENOYLCOARED-RXN | 1/1 Saturated-Fatty-Acyl-CoA+ 1/1 NADP-> 1/1 TRANS-D2-ENOYL-COA+ 1/1 NADPH |
| RXN-8001 | 2/1 NAD+ 1/1 HISTIDINOL-> 1/1 HIS+ 2/1 NADH |
| MAL-DEH-GLYOX-RXN | 1/1 MAL+ 1/1 NAD-> 1/1 PROTON+ 1/1 OXALACETIC_ACID+ 1/1 NADH |
| RXN-9951 | 1/1 THREO-DS-ISO-CITRATE+ 1/1 NADP-> 1/1 OXALO-SUCCINATE+ 1/1 NADPH+ 1/1 PROTON |
| RXN0-1147 | 1/1 SUC-COA+ 1/1 Oxo-glutarate-dehydrogenase-DH-lipoyl<> 1/1 CO-A+ 1/1 Oxo-glutarate-dehydro-suc-DH-lipoyl |
| MANNKIN-RXN | 1/1 MANNOSE+ 1/1 ATP-> 1/1 PROTON+ 1/1 MANNOSE-6P+ 1/1 ADP |
| 2.5.1.51-RXN | 1/1 ACETYLSERINE+ 1/1 PYRAZOLE-> 1/1 PROTON+ 1/1 CPD-670+ 1/1 ACET |
| RXN-7461 | 1/1 ACETYLSERINE+ 1/1 DIHYDROXYPIRIDIN-CPD-> 1/1 AMINO-ETCETERA-PYRIDIN-1-YL-PROPANOATE+ 2/1 PROTON+ 1/1 ACET |
| RXN-6622 | 1/1 CYS-GLY+ 1/1 WATER-> 1/1 CYS+ 1/1 GLY |
| CYSTAGLY-RXN | 1/1 L-CYSTATHIONINE+ 1/1 WATER<> 1/1 PROTON+ 1/1 AMMONIA+ 1/1 2-OXOBUTANOATE+ 1/1 CYS |
| LCYSDESULF-RXN | 1/1 CYS+ 1/1 WATER-> 1/1 AMMONIA+ 1/1 PYRUVATE+ 1/1 HS+ 1/1 PROTON |
| GMP-SYN-NH3-RXN | 1/1 AMMONIA+ 1/1 XANTHOSINE-5-PHOSPHATE+ 1/1 ATP-> 1/1 AMP+ 1/1 PPI+ 1/1 GMP |
| 1.8.4.1-RXN | 1/1 HOMOCYSTINE+ 2/1 GLUTATHIONE-> 1/1 OXIDIZED-GLUTATHIONE+ 2/1 HOMO-CYS |
| GCVT-RXN | 1/1 AMINOMETHYLDIHYDROLIPOYL-GCVH+ 1/1 THF-> 1/1 DIHYDROLIPOYL-GCVH+ 1/1 METHYLENE-THF+ 1/1 AMMONIA |
| 3.6.3.44-RXN | 1/1 Xenobiotic + 1/1 WATER + 1/1 ATP -> 1/1 Xenobiotic + 1/1 Pi + 1/1 ADP |
| 3.6.3.6-RXN | 1/1 PROTON + 1/1 WATER + 1/1 ATP -> 1/1 PROTON + 1/1 Pi + 1/1 ADP |
| 3.6.3.8-RXN | 1/1 CA+2 + 1/1 WATER + 1/1 ATP -> 1/1 CA+2 + 1/1 Pi + 1/1 ADP |
| 3.6.4.1-RXN | 1/1 WATER + 1/1 ATP -> 1/1 PROTON + 1/1 Pi + 1/1 ADP |
| RXN-11135 | 1/1 ATP + 1/1 WATER -> 1/1 PROTON + 1/1 ADP + 1/1 Pi |
| RXN-11109 | 1/1 ATP + 1/1 WATER -> 1/1 PROTON + 1/1 ADP + 1/1 Pi |
| 3.6.4.3-RXN | 1/1 WATER + 1/1 ATP -> 1/1 PROTON + 1/1 Pi + 1/1 ADP |
| 3.6.3.1-RXN | 1/1 Phospholipids + 1/1 ATP + 1/1 WATER -> 1/1 Phospholipids + 1/1 ADP + 1/1 Pi + 2/1 PROTON |
| ATPSYN-RXN | 3/1 PROTON + 1/1 WATER + 1/1 ATP <> 4/1 PROTON + 1/1 Pi + 1/1 ADP |
| 3.6.3.3-RXN | 1/1 CD+2 + 1/1 WATER + 1/1 ATP -> 1/1 CD+2 + 1/1 Pi + 1/1 ADP |
| 3.6.3.5-RXN | 1/1 ZN+2 + 1/1 WATER + 1/1 ATP -> 1/1 ZN+2 + 1/1 Pi + 1/1 ADP |
| METBALT-RXN | 2/1 PROTON + 1/1 AMMONIA + 1/1 2-OXOBUTANOATE + 1/1 SUC -> 1/1 O-SUCCINYL-L-HOMOSERINE + 1/1 WATER |
| 2.7.4.22-RXN | 1/1 ATP + 1/1 UMP <> 1/1 ADP + 1/1 UDP |
| RXN1F-146 | 1/1 CPD1F-98 -> 1/1 CPD1F-114 + 4/1 PROTON |
| RXN-6283 | 1/1 FORMYL-THF-GLU-N + 1/1 ADP + 1/1 Pi -> 1/1 THF-GLU-N + 1/1 ATP + 1/1 FORMATE |
| MYOSIN-LIGHT-CHAIN-PHOSPHATASE-RXN | 1/1 WATER + 1/1 CPD-8563 -> 2/1 PROTON + 1/1 Pi + 1/1 CPD-8564 |
| RXN-9217 | 1/1 PROTON + 1/1 ALL-TRANS-HEXAPRENYL-DIPHOSPHATE + 1/1 DIHYDROXYNAPHTHOATE -> 1/1 CPD-12116 + 1/1 PPI + 1/1 CARBON-DIOXIDE |
| RXN-9358 | 1/1 CPD-9610 + 1/1 DIHYDROXYNAPHTHOATE + 1/1 PROTON -> 1/1 CPD-12119 + 1/1 PPI + 1/1 CARBON-DIOXIDE |
| DCTP-DEAM-RXN | 1/1 WATER + 1/1 DCTP -> 1/1 DUTP + 1/1 AMMONIA |
| BUTYRATE-KINASE-RXN | 1/1 "BUTYRIC_ACID" + 1/1 "ATP" <> 1/1 "BUTYRYL-P" + 1/1 "ADP" |
| ACETYLHOMOSER-CYS-RXN | 1/1 "CPD-667" + 1/1 "HS" -> 1/1 "HOMO-CYS" + 1/1 "ACET" |
| PHOSPHATE-BUTYRYLTRANSFERASE-RXN | 1/1 "BUTYRYL-COA" + 1/1 "Pi" <> 1/1 "BUTYRYL-P" + 1/1 "CO-A" |
| R11-RXN | 1/1 "BUTYRYL-COA" + 1/1 "ACET" <> 1/1 "ACETYL-COA" + 1/1 "BUTYRIC_ACID" |
| 1.8.4.8-RXN | 1.0 "Red-Thioredoxin" + 1.0 "PAPS" -> 1.0 "PAP" + 1.0 "SO3" + 1.0 "Ox-Thioredoxin" |
| PREPHENATE-DEHYDROGENASE-(NADP)-RXN | 1/1 "NADP" + 1/1 "PREPHENATE" -> 1/1 "NADPH" + 1/1 "P-HYDROXY-PHENYLPYRUVATE" + 1/1 "CARBON-DIOXIDE" |
| PROPIONATE-COA-TRANSFERASE-RXN | 1/1 "ACETYL-COA" + 1/1 "PROPIONATE" -> 1/1 "PROPIONYL-COA" + 1/1 "ACET" |
