# Supplementary material for: Reconstruction of Oryza sativa indica Genome Scale Metabolic Model and Its Responses to Varying RuBisCO Activity, Light Intensity, and Enzymatic Cost Conditions
Source: Front Plant Sci. 2017 Nov 30;8:2060. doi: 10.3389/fpls.2017.02060 (PMC5715477; doi:10.3389/fpls.2017.02060)
Supplement: Supplementary file 2 [file Table_2.DOC]

**Table S2.** List of reactions added in the model. 125 reactions were added in the model.

| **Reaction Name** | **Reaction** |
| --- | --- |
| **#Source: Brenda/Metacyc** |  |
|  |  |
| DIAMINOPIMDECARB-RXN | 1/1 MESO-DIAMINOPIMELATE -> 1/1 CARBON-DIOXIDE + 1/1 LYS |
| CYSPH-RXN | 1/1 O-PHOSPHO-L-HOMOSERINE + 1/1 CYS -> 1/1 L-CYSTATHIONINE + 1/1 Pi |
| HOMOCYSMETB12-RXN | 1/1 HOMO-CYS + 1/1 5-METHYL-THF -> 1/1 THF + 1/1 MET |
| MALYL-COA-LYASE-RXN | 1/1 CPD-208 -> 1/1 ACETYL-COA + 1/1 GLYOX |
| SULFITE-REDUCTASE-(FERREDOXIN)-RXN | 1/1 SO3 + 3/1 Reduced-ferredoxins -> 3/1 Oxidized-ferredoxins + 1/1 HS |
| SO3OXTFER-RXN | 2/1 Cytochromes-C-Reduced + 1/1 SULFATE -> 1/1 SO3 + 2/1 Cytochromes-C |
| RXN-7800 | 1/1 CPD-7100 <> 1/1 2K-4CH3-PENTANOATE + 1/1 CARBON-DIOXIDE |
| ATPPHOSPHORIBOSYLTRANS-RXN | 1/1 PPI + 1/1 PHOSPHORIBOSYL-ATP <> 1/1 PRPP + 1/1 ATP |
| GLUTAMIDOTRANS-RXN | 1/1 PHOSPHORIBULOSYL-FORMIMINO-AICAR-P + 1/1 GLN -> 1/1 D-ERYTHRO-IMIDAZOLE-GLYCEROL-P + 1/1 GLT + 1/1 AICAR |
| 1.4.3.22-Rxn | CPD-313 + OXYGEN-MOLECULE + WATER -> CPD-6082 + AMMONIA + HYDROGEN-PEROXIDE |
| SPERMINE-SYNTHASE-RXN | 1/1 S-ADENOSYLMETHIONINAMINE + 1/1 SPERMIDINE -> 1/1 5-METHYLTHIOADENOSINE + 1/1 SPERMINE |
| PROPIONATE-COA-LIGASE | PROPIONATE + ATP + CO-A -> PROPIONYL-COA + ADP + PPI + PROTON |
| LACTOYL-COA-DEHYDRATASE-RXN | 1/1 D-LACTOYL-COA <> 1/1 ACRYLYL-COA + WATER |
| 3-HYDROXYPROPIONATE-DEHYDROGENASE-RXN | 1/1 3-HYDROXY-PROPIONATE + 1/1 NAD <> 1/1 NADH + 1/1 MALONATE-S-ALD |
| 2.6.1.18-RXN | 1/1 L-ALPHA-ALANINE + 1/1 MALONATE-S-ALD <> 1/1 PYRUVATE + 1/1 B-ALANINE |
| AIRCARBOXY-RXN | 1/1 PHOSPHORIBOSYL-CARBOXY-AMINOIMIDAZOLE <> 1/1 CARBON-DIOXIDE + 1/1 5-PHOSPHORIBOSYL-5-AMINOIMIDAZOLE |
| RXN0-745 | 1/1 ATP <> 1/1 DATP |
| RXN0-384 | 1/1 WATER + 1/1 DATP -> 1/1 PPI + 1/1 DAMP |
| GMP-REDUCT-RXN | 1/1 AMMONIA + 1/1 NADP + 1/1 IMP -> 1/1 NADPH + 1/1 GMP |
| GMKALT-RXN | 1/1 DGMP + 1/1 ATP <> 1/1 DGDP + 1/1 ADP |
| RXN0-746 | 1/1 GTP <> 1/1 DGTP |
| DIHYDROOROTOX-RXN | 1/1 DI-H-OROTATE + 1/1 OXYGEN-MOLECULE -> 1/1 HYDROGEN-PEROXIDE + 1/1 OROTATE |
| UMPKI-RXN | 1/1 UMP + 1/1 ATP <> 1/1 UDP + 1/1 ADP |
| CMPKI-RXN | 1/1 CMP + 1/1 ATP <> 1/1 CDP + 1/1 ADP |
| RXN-7913 | 1/1 DCMP + 1/1 ATP <> 1/1 ADP + 1/1 DCDP |
| RXN-1101 | 1/1 NADPH + 1/1 COUMAROYL-COA -> 1/1 COUMARALDEHYDE + 1/1 NADP + 1/1 CO-A |
| RXN-1103 | 1/1 NADPH + 1/1 COUMARATE + 1/1 OXYGEN-MOLECULE -> 1/1 CAFFEATE + 1/1 NADP |
| RXN-1106 | 1/1 NADPH + 1/1 FERULOYL-COA -> 1/1 CONIFERYL-ALDEHYDE + 1/1 NADP + 1/1 CO-A |
| RXN-1143 | 1/1 S-ADENOSYLMETHIONINE + 1/1 CONIFERYL-ALDEHYDE -> 1/1 ADENOSYL-HOMO-CYS + 1/1 SINAPALDEHYDE |
| SULFATE-ADENYLYLTRANSFERASE-ADP-RXN | 1/1 SULFATE + 1/1 ADP -> 1/1 APS + 1/1 Pi |
| SPONTPRO-RXN | 1/1 L-GLUTAMATE_GAMMA-SEMIALDEHYDE <> 1/1 l-delta(1)-pyrroline_5-carboxylate |
| 2.4.1.32-RXN | 1/1 GDP-MANNOSE + 1/1 Glucomannans_in -> 1/1 GDP + 1/1 Glucomannans_out |
| ACETYL_COA_SYN | ACET + ATP + CO-A -> ACETYL-COA + AMP + PPI |
| LACCOA-RXN | 1/1 PROPIONYL-COA + 1/1 D-LACTATE -> 1/1 D-LACTOYL-COA + 1/1 PROPIONATE |
| RXN-6383 | 1/1 ACRYLYL-COA <> 1/1 3-HYDROXY-PROPIONYL-COA |
| Acyl-Coa-Dehydrogenase | PROPIONYL-COA + FAD + PROTON -> ACRYLYL-COA + FADH2 |
| ARP_SYN_RXN | 1/1 CPD-1086 -> 1/1 ARP |
| DIMETHYL-D-RIBITYL-LUMAZINE-SYN | 1/1 ARP + 1/1 DIHYDROXY-BUTANONE-P -> 1/1 DIMETHYL-D-RIBITYL-LUMAZINE |
| AMINO-RIBOSYLAMINO-1H-3H-PYR-DIONE_RXN | 1/1 AMINO-RIBOSYLAMINO-1H-3H-PYR-DIONE + 1/1 DIHYDROXY-BUTANONE-P -> 1/1 DIMETHYL-D-RIBITYL-LUMAZINE |
| FAD_reduction_RXN | 1/1 FAD + 2/1 PROTON <> 1/1 FADH2 |
| THR-DEAMINASE-RXN  ALLENE-OXIDE-SYNTHASE-RXN  ALLENE-OXIDE-CYCLASE-RXN  12-OXOPHYTODIENOATE-REDUCTASE-RXN  ISOJASMONATE-RXN  JASMONATE-SYN-RXN  3-HYDROXYACYL-COA-DEHYDROGENASE-RXN | 1/1 THR -> 1/1 2-OXOBUTANOATE + 1/1 AMMONIA  1/1 "13-HYDROPEROXYOCTADECA-911-DIENOATE" -> 1/1 "12,13(S)-epoxylinolenate"  1/1 "12,13(S)-epoxylinolenate" -> 1/1 "12-oxo-cis-10,15-phytodienoate"  1/1 "12-oxo-cis-10,15-phytodienoate" + 1/1 "NADPH" -> 1/1 "3-oxo-2-(cis-2'-pentenyl)-cyclopentane-1-octanoate" + 1/1 "NADP"  1/1 "3-oxo-2-(cis-2'-pentenyl)-cyclopentane-1-octanoate" + 3/1 "OXYGEN-MOLECULE" -> 1/1 "7-isojasmonate" + 3/1 "ACET"  1/1 "7-isojasmonate" -> 1/1 "Jasmonate"  1.0 "D-3-HYDROXYACYL-COA" + 1/1 "NAD" <> 1.0 "CPD-11524" + 1/1 "NADH" |
| **#Ref: Chatterjee and Kundu,2015; Bauwe et al., 2010; Jimenez et al, 1997** |  |
| chl_LightCyc | 6/1 ADP_str + 14/1 Photon + 6/1 Pi_str -> 6/1 ATP_str |
| chl_LightNonCyc | 9/2 ADP_str + 7/2 NADP_str + 9/2 Pi_str + 14/1 Photon + 7/2 WATER -> 7/4 O2_str + 7/2 x_Proton_str + 7/2 NADPH_str + 9/2 ATP_str |
| chl_Rubisco | CO2_str + 1 RuBP_str -> 2 PGA_str |
| chl_RuBPOxid | O2_str + RuBP_str -> PGA_str + PGly_str |
| chl_G1P_ADPTrans | G1P_str + ATP_str -> 1.0 PPI_str + ADPG_str |
| chl_StSynth | ADPG_str -> ADP_str + Starch_str |
| chl_StPase | Starch_str + Pi_str -> G1P_str |
| chl_PG_rxn | PGly_str + 1/1 WATER -> 1/1 GLYCOLLATE_str + Pi_str |
| chl_RXN7658 | 1.0 NADPH_str + 1.0 GERANYLGERANYL-PP_str <> 1.0 NADP_str + 1.0 CPD-7002_str |
| chl_RXN7659 | 1.0 NADPH_str + 1.0 CPD-7002_str <> 1.0 NADP_str + 1.0 CPD-7003_str |
| chl_RXN7660 | 1.0 NADPH_str + 1.0 CPD-7003_str <> 1.0 NADP_str + 1.0 PHYTYL-PYROPHOSPHATE_str |
| chl_RXNMGPROTOPORPHYRINMETHYLESTERSYN | 1.0 MG-PROTOPORPHYRIN_str + 1.0 S-ADENOSYLMETHIONINE_str -> 1.0 MG-PROTOPORPHYRIN-MONOMETHYL-ESTER_str + 1.0 ADENOSYL-HOMO-CYS_str |
| chl_RXN5283 | 1.0 NADPH_str + 1.0 O2_str + 1.0 13-HYDROXY-MAGNESIUM-PROTOPORP_str -> 1.0 NADP_str + 1.0 131-OXO-MAGNESIUM-PROTOPORPHYRIN-IX-13-M_str |
| chl_RXN5284 | 1.0 NADPH_str + 1.0 O2_str + 1.0 131-OXO-MAGNESIUM-PROTOPORPHYRIN-IX-13-M_str -> 1.0 NADP_str + 1.0 DIVINYL-PROTOCHLOROPHYLLIDE-A_str |
| chl_RXN1F72 | 1.0 NADPH_str + 1.0 DIVINYL-PROTOCHLOROPHYLLIDE-A_str -> 1.0 NADP_str + 1.0 MONO-VINYL-PROTOCHLOROPHYLLIDE-A_str |
| chl_RXN1F10 | 1.0 NADPH_str + 1.0 MONO-VINYL-PROTOCHLOROPHYLLIDE-A_str -> 1.0 NADP_str + 1.0 CHLOROPHYLLIDE-A_str |
| chl_RXN1F66 | 1.0 PHYTYL-PYROPHOSPHATE_str + 1.0 CHLOROPHYLLIDE-A_str -> 1.0 PPI_str + 1.0 CHLOROPHYLL-A_str |
| mit_PyrDH | Pyr + NAD_mit + CoA -> AcCoA + NAD_mitH + mit_CO2 + Proton |
| mit_CitSynth | OxalAc + AcCoA + WATER <> Citrate + CoA |
| mit_AconDHatase | Citrate <> CisAconitate |
| mit_AconHydr | CisAconitate <> IsoCitrate |
| mit_AlphaKGDH | AlphaKG + NAD_mit + CoA <> SucCoA + NAD_mitH + Proton + mit_CO2 |
| mit_AOX | 2 QH2 + mit_O2-> 2 Q + 2 WATER |
| mit_Complex_IV | 4 Cyt_red + 8 Proton + mit_O2 -> 4 Cyt_ox + 2 WATER + 4 PROTON |
| mit_Complex_V | 3 PROTON + mit_ADP + mit_Pi -> 3 Proton + mit_ATP |
| mit_GCVMULTI | 1/1 mit_THF + 1/1 NAD_mit + 1/1 mit_GLY -> 1/1 mit_METHYLENE-THF + 1/1 NAD_mitH +1/1 mit_AMMONIA + 1/1 mit_CO2 |
| per_RXN-3523 | 2/1 per_CPD-318 -> 1/1 per_L-DEHYDRO-ASCORBATE + 1/1 per_ASCORBATE |
| per_1.8.5.1-RXN | 1/1 per_L-DEHYDRO-ASCORBATE + 2/1 per_GLUTATHIONE -> 1/1 per_OXIDIZED-GLUTATHIONE + 1/1 per_ASCORBATE |
| per_GLYCERATEDEHYDROGENASERXN | 1/1 per_Proton + 1/1 per_OH-PYR + 1/1 per_NADH -> 1/1 per_NAD + 1/1 per_GLYCERATE |
| per_GLYCINEAMINOTRANSFERASERXN | 1/1 per_GLT + 1/1 per_GLYOX -> 1/1 per_2-KETOGLUTARATE + 1/1 per_GLY |
| **##transporters** |  |
| chl_glyco_glycerate_tx | 2 GLYCOLLATE_str + GLYCERATE <> 2 GLYCOLLATE + GLYCERATE_str |
| chl_Mal2OG_tx | MAL_str + 2-KETOGLUTARATE <> 2-KETOGLUTARATE_str + MAL |
| chl_MalGLT_tx | MAL_str + GLT <> GLT_str + MAL |
| chl_Gln_Glt_tx | GLT + GLN_str <> GLN + GLT_str |
| chl_NITRATE_tx | NITRATE <> NITRATE_str |
| chl_Photon_tx | x_Photon -> Photon |
| chl_CO2_tx | CARBON-DIOXIDE -> CO2_str |
| chl_O2_tx | OXYGEN-MOLECULE <> O2_str |
| chl_GAP_tx | GAP + Pi_str <> GAP_str + Pi |
| chl_DHAP_tx | DIHYDROXY-ACETONE-PHOSPHATE + Pi_str <> DHAP_str + Pi |
| chl_E4P_tx | ERYTHROSE-4P + Pi_str <> E4P_str + Pi |
| chl_Ru5P_tx | RIBULOSE-5P + Pi_str <> R5P_str + Pi |
| chl_G6P_tx | GLC-6-P + Pi_str <> G6P_str + Pi |
| chl_Star_tx | Starch_str <> 1-4-alpha-D-Glucan |
| chl_PYR_tx | PYRUVATE <> PYR_str |
| chl_PEP_tx | PHOSPHO-ENOL-PYRUVATE + Pi_str <> PEP_str + Pi |
| chl_MalOxAc_tx | MAL_str + OXALACETIC_ACID <> MAL + OAA_str |
| chl_PGA_tx | G3P + Pi_str <> PGA_str + Pi |
| chl_AMMONA_tx | AMMONIA <> AMMONIA_str |
| chl_ammonia_tx | x_NH3 -> AMMONIA_str |
| chl_S1_tx | S-ADENOSYLMETHIONINE <> S-ADENOSYLMETHIONINE_str |
| chl_S2_tx | ADENOSYL-HOMO-CYS_str <> ADENOSYL-HOMO-CYS |
| per_OXYGENMOLECULE_tx | 1/1 per_OXYGEN-MOLECULE <> 1/1 OXYGEN-MOLECULE |
| per_OHPYR_tx | 1/1 per_OH-PYR -> 1/1 OH-PYR |
| per_2KETOGLUTARATE_tx | 1/1 per_2-KETOGLUTARATE <> 1/1 2-KETOGLUTARATE |
| per_GLT_tx | 1/1 per_GLT <> 1/1 GLT |
| per_SUPER-OXIDE_tx | 1/1 per_SUPER-OXIDE <> 1/1 SUPER-OXIDE |
| per_OAA_tx | 1/1 per_OXALACETIC_ACID <> 1/1 OXALACETIC_ACID |
| per_GLYCERATE_tx | 1/1 per_GLYCERATE <> 1/1 GLYCERATE_str |
| per_GLY_SER_tx | 1/1 SER + 2/1 per_GLY -> 2/1 GLY + 1/1 per_SER |
| per_GLYCOLLATE_tx | GLYCOLLATE_str <> per_GLYCOLLATE |
| per_LALPHAALANINE_tx | 1/1 per_L-ALPHA-ALANINE <> 1/1 L-ALPHA-ALANINE |
| per_PYRUVATE_tx | 1/1 per_PYRUVATE <> 1/1 PYRUVATE |
| per_Mal_tx | 1/1 per_MAL <> 1/1 MAL |
| mit_SER_tx | SER <> mit_SER |
| mit_GLY_tx | GLY <> mit_GLY |
| mit_Cit_tx | CIT <> Citrate |
| mit_AKG_tx | 2-KETOGLUTARATE <> AlphaKG |
| mit_Suc_tx | SUC <> Suc |
| mit_Fum_tx | FUM <> Fum |
| mit_Mal_tx | MAL <> Mal |
| mit_OAA_tx | OXALACETIC_ACID <> OxalAc |
| mit_Pyr_tx | PYRUVATE -> Pyr |
| mit_CO2_tx | CARBON-DIOXIDE <> mit_CO2 |
| mit_O2_tx | OXYGEN-MOLECULE <> mit_O2 |
| mit_am_tx | mit_AMMONIA <> AMMONIA |
| mit_Proton_Pi_tx | Pi + PROTON <> mit_Pi + Proton |
| mit_ATP_tx | ADP + mit_ATP <> ATP + mit_ADP |

Compartments: chl_ , chloroplast; per_ , peroxisome; mit_ , mitochondrion.
