# Supplementary material for: Reconstruction of Oryza sativa indica Genome Scale Metabolic Model and Its Responses to Varying RuBisCO Activity, Light Intensity, and Enzymatic Cost Conditions
Source: Front Plant Sci. 2017 Nov 30;8:2060. doi: 10.3389/fpls.2017.02060 (PMC5715477; doi:10.3389/fpls.2017.02060)
Supplement: Supplementary file 3 [file Data_Sheet_1.DOCX]

Supplementary Material

# Reconstruction of *Oryza sativa indica* genome scale metabolic model and its responses to varying RubisCO activity, light intensity and enzymatic cost conditions

**Ankita Chatterjee, Benazir Huma, Rahul Shaw**

* Correspondence: skbmbg@caluniv.ac.in

**1 Supplementary Data**

Supplementary Data S1- Uniprot reaction ids and GPR association for the reactions in the model.

Supplementary Data S2 - Model files in SBML and Excel sheet.

Supplementary Data S3 – Reactions unique to *indica* Model

Supplementary Data S4 – Results of FVA.

# Supplementary Tables

Supplementary Table S1 – List of reactions removed from the draft model.

Supplementary Table S2 – List of reactions added to the draft model.
